# Effects of Age and Estrogen on Skeletal Gene Expression in Humans as Assessed by RNA Sequencing

**DOI:** 10.1371/journal.pone.0138347

**Published:** 2015-09-24

**Authors:** Joshua N. Farr, Matthew M. Roforth, Koji Fujita, Kristy M. Nicks, Julie M. Cunningham, Elizabeth J. Atkinson, Terry M. Therneau, Louise K. McCready, James M. Peterson, Matthew T. Drake, David G. Monroe, Sundeep Khosla

**Affiliations:** 1 Division of Endocrinology, Department of Medicine, Mayo Clinic College of Medicine, Rochester, MN, 55905, United States of America; 2 Robert and Arlene Kogod Center on Aging, Mayo Clinic College of Medicine, Rochester, MN, 55905, United States of America; 3 Department of Experimental Pathology and Laboratory Medicine, Mayo Clinic College of Medicine, Rochester, MN, 55905, United States of America; 4 Division of Biomedical Statistics and Informatics, Department of Health Sciences Research, Mayo Clinic College of Medicine, Rochester, MN, 55905, United States of America; Universidade de São Paulo, BRAZIL

## Abstract

Precise delineation of the specific genes and pathways altered with aging and estrogen (E) therapy may lead to new skeletal biomarkers and the development of novel bone therapeutics. Previous human bone studies, however, have been limited by only examining pre-specified genes and pathways. High-throughput RNA sequencing (RNAseq), on the other hand, offers an unbiased approach to examine the entire transcriptome. Here we present an RNAseq analysis of human bone samples, obtained from iliac crest needle biopsies, to yield the first *in vivo* interrogation of all genes and pathways that may be altered in bone with aging and E therapy in humans. 58 healthy women were studied, including 19 young women (mean age ± SD, 30.3 ± 5.4 years), 19 old women (73.1 ± 6.6 years), and 20 old women treated with 3 weeks of E therapy (70.5 ± 5.2 years). Using generally accepted criteria (false discovery rate [*q*] < 0.10), aging altered a total of 678 genes and 12 pathways, including a subset known to regulate bone metabolism (e.g., Notch). Interestingly, the *LEF1* transcription factor, which is a classical downstream target of the Wnt/β-catenin signaling pathway, was significantly downregulated in the bones from the old versus young women; consistent with this, *LEF1* binding sites were significantly enriched in the promoter regions of the differentially expressed genes in the old versus young women, suggesting that aging was associated with alterations in Wnt signaling in bone. Further, of the 21 unique genes altered in bone by E therapy, the expression of *INHBB* (encoding for the inhibin, beta B polypeptide), which decreased with aging (by 0.6-fold), was restored to young adult levels in response to E therapy. In conclusion, our data demonstrate that aging alters a substantial portion of the skeletal transcriptome, whereas E therapy appears to have significant, albeit less wide-ranging effects. These data provide a valuable resource for the potential identification of novel biomarkers associated with age-related bone loss and also highlight potential pathways that could be targeted to treat osteoporosis.

**Trial Registration:**

ClinicalTrials.gov NCT02349113

## Introduction

Aging is the single largest risk factor for bone loss in both sexes [1]. While virtually all current therapies target osteoclast-mediated bone resorption, age-related bone loss results, in large part, from a defect in the number and/or function of osteoblasts—the cells within basic multicellular units (BMUs) responsible for forming new bone. Thus, reflecting the age-related defect in bone formation, histologically measured mean wall thickness, a measure of the work done by osteoblasts in BMUs, declines with age in both sexes [2]. However, while serum bone formation markers steadily decline with age in men [3,4], they generally increase in older women [4]. This is because the marked estrogen (E) deficiency in postmenopausal women leads to an overall increase in bone turnover, resulting in more BMUs, even though there is a relative reduction in bone formation at the cellular level [5]. Ultimately, this imbalance between bone resorption and formation leads to net bone loss. Therefore, impaired bone formation is a hallmark of age-related bone loss in both sexes.

Despite this understanding, directly identifying the underlying mechanisms for impaired bone formation with aging and E deficiency in humans is a significant gap in knowledge. Accordingly, the results of studies aimed at these issues may lead to novel approaches to prevent or reverse age-related bone loss. In addition, such studies may lead to the identification of new skeletal biomarkers to permit better targeting of therapies to individual patients. However, the specific genes and pathways in human bone that are regulated by aging or E remain unclear. These genes and pathways must be defined more precisely in order to develop novel therapeutic approaches to combat age-related bone loss.

To address this issue, we have developed and validated an approach to obtain and analyze small needle bone biopsies (1–2 mm diameter) from the posterior iliac crest of humans [6,7]. Using this approach, we have to date obtained bone samples from 60 women, including 20 young women as well as 40 old women (20 per group) receiving either no therapy or 3 weeks of short-term E therapy. Previously, we coupled this methodology to customized, in-house quantitative polymerase chain reaction (QPCR) analyses of nearly 300 genes related to bone metabolism in this cohort of women [6,7]. A limitation of these studies, however, was that we only examined pre-specified pathways and genes using QPCR. High-throughput RNA sequencing (RNAseq), on the other hand, offers an unbiased approach to examine the entire transcriptome. Here we present a high-throughput RNAseq analysis of our previously characterized human bone samples [6,7] to yield the first *in vivo* interrogation of all potential genes and pathways in bone that may be altered with aging and in response to E therapy in women.

## Materials and Methods

### Study subjects

This study was approved by the Mayo Clinic Institutional Review Board (IRB), and full informed written consent was obtained from all subjects. As described previously [6,7], we recruited a total of 60 healthy women, including 20 young women (mean age ± SD, 30.0 ± 5.4 years, range 22 to 40 years) as well as 40 old women (n = 20 per group) receiving either no therapy (72.9 ± 6.5 years, 65 to 88 years) or 3 weeks of E therapy (70.5 ± 5.2 years, 65 to 88 years). All old women were ≥65 years of age and postmenopausal (i.e., absence of menses >1 year; serum follicle stimulating hormone [FSH] >20 IU/L), whereas all young women were premenopausal. Old women receiving E were treated with 100 μg/d of transdermal 17β-estradiol (Alora, Watson Pharma Inc., Corona, CA); patches were changed every 3–4 days for the 3 week study duration. All potential subjects were rigorously screened for coexisting disease and excluded if they had low body stores of vitamin D (serum total 25-hydroxyvitamin D [25(OH)D] of <20 ng/ml), any clinical abnormalities in other serum biochemistries (i.e., calcium, phosphorus, alkaline phosphatase, aspartate transaminase, creatinine, parathyroid hormone [PTH], thyroid stimulating hormone [TSH], and FSH), or any disorders associated with altered skeletal structure or function. This included the presence of chronic renal impairment, chronic liver disease, severe neuropathic disease, unstable cardiovascular disease, malignancy, chronic gastrointestinal disease, hypo- or hyperparathyroidism, acromegaly, Cushing’s syndrome, hypopituitarism, severe chronic obstructive pulmonary disease, alcoholism, or diabetes. In addition, subjects with a history of pathological fractures (e.g., due to Paget’s disease, myeloma, metastatic malignancy) were excluded. Further, subjects were excluded if undergoing treatment with any medication that can affect bone metabolism such as corticosteroids (>3 months at any time or >10 days within the previous year), anticonvulsant therapy (within the previous year), pharmacological doses of thyroid hormone (causing TSH to decline below normal), adrenal or anabolic steroids, aromatase inhibitors, calcitonin, calcium supplementation >1200 mg/d (within the preceding 3 months), bisphosphonates, denosumab, E or selective E receptor modulator (within the past year), sodium fluoride, or teriparatide. Clinical details in the medical records were reviewed to determine if subjects met study criteria. These details were confirmed during an interview with each subject.

**Fig 1:**
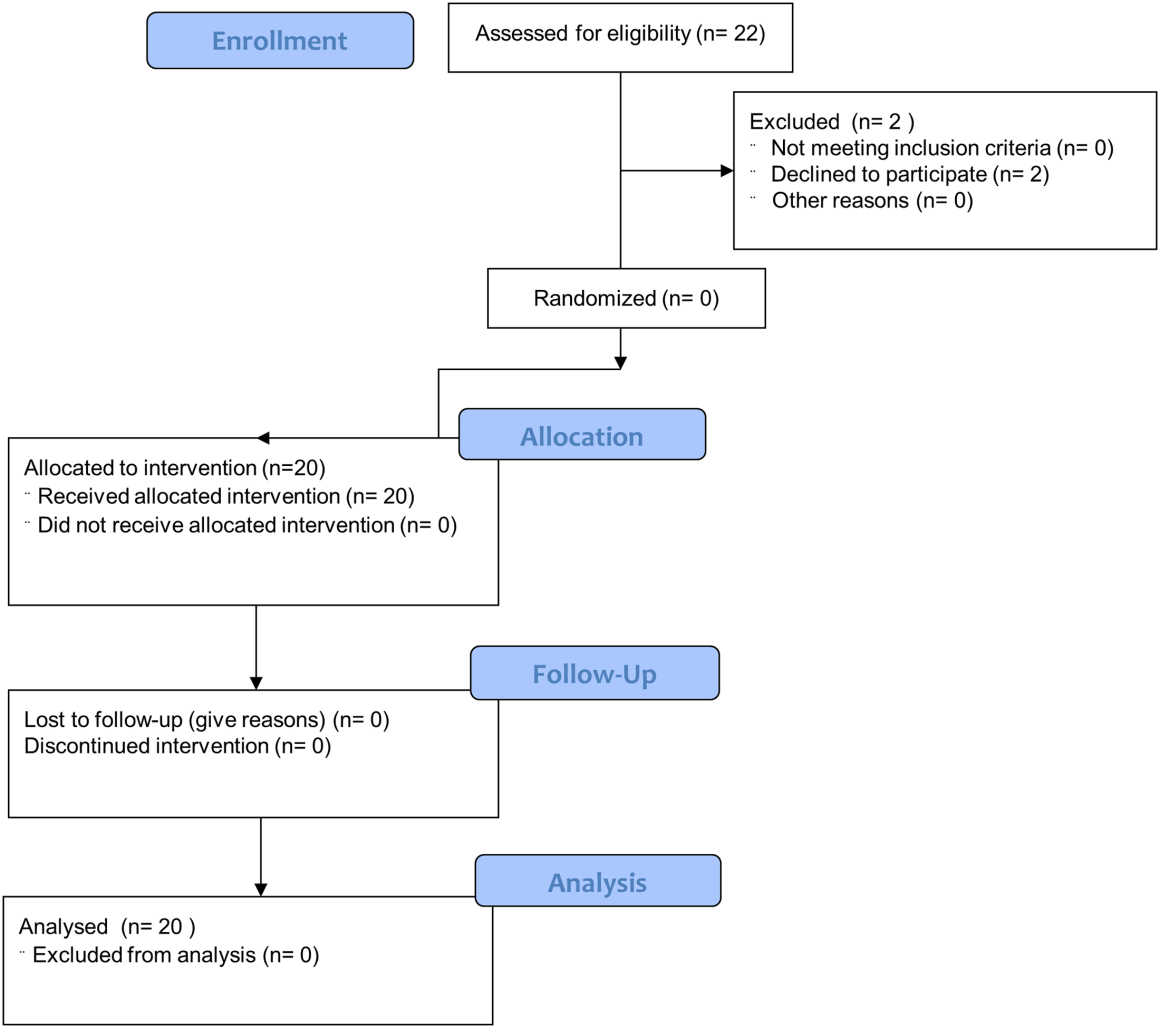
Flow diagram for enrollment and allocation of the subjects in the E arm of the study.

The young and untreated old women were studied under a common IRB protocol. The women treated with E were studied under a separate IRB protocol; since this group received an intervention, it is now considered a clinical trial. As such, this component of the study was registered at ClinicalTrials.gov (registration number NCT02349113). The study was not registered before the enrollment of patients started because when this study enrolled patients (2011–2012), the requirements for registration of all studies with an intervention, even for non-therapeutic (i.e., mechanistic) studies, were less stringent. The authors do confirm that all ongoing and related trials for this drug/intervention are registered. Subjects were recruited and studied between February 1, 2011 and September 13, 2012. A flowchart for enrollment and allocation of the subjects in the E arm of the study is provided in Fig 1.

### Study protocol

All studies were performed and samples collected in the outpatient Clinical Research Unit at the Mayo Clinic (Rochester, Minnesota, USA) with study subjects maintaining their usual diet and calcium intake. After an overnight fast, morning blood was drawn from potential candidates and serum was analyzed for determination of serum biochemistries (see *Study subjects*). Blood samples were stored at -80°C. Screening procedures also included a medical history and physical examination. Anthropometric data were collected on all subjects wearing light-weight clothing and no shoes. Weight was obtained using an electronic scale (Model 5002, Tronic, Inc., White Plains, NY) and height was measured using a customized stadiometer (Mayo Section of Engineering). Body mass index (BMI, kg/m^2^) was calculated as the ratio of weight to height^2^. Small needle biopsies of bone were obtained from all subjects as detailed below.

### Obtaining and processing needle biopsies of bone

Using an approach slightly modified from that used clinically by hematologists to obtain bone marrow aspirates and biopsies, we obtained small needle bone biopsies from the posterior iliac crest of all subjects using an 8G needle under local anesthesia (1% lidocaine) and monitored intravenous (IV) sedation (1–3 mg of IV midazolam and 50–100 μg of fentanyl), as described previously [6,7]. All 60 biopsies were performed without any complications. Each biopsy is 1–2 mm in width and ~1–2 cm in length, and contains both cortical and trabecular bone (see [8] for a μCT analysis of a similar bone sample). Of note, we found that the standard practice of snap freezing and storing the bone samples for subsequent homogenization results in significant RNA degradation, with higher variability in RNA integrity number (RIN) values. Thus, we immediately homogenized (Tissue Tearor, Cole-Parmer) the bone samples in lysis buffer (QIAzol; QIAGEN) and subsequently stored them at -80°C until the time of further processing. This procedure permitted isolation of intact RNA (RIN values consistently >9) from 58 of the 60 (19 young, 19 old, and 20 E therapy) bone biopsy samples; these 58 samples were included in the RNAseq analysis of this report.

### RNA isolation and whole transcriptome RNA sequencing (RNAseq)

As described previously [6,7,8], total RNA was isolated using the RNeasy Micro Kit (Qiagen, Valencia, CA) and treated with the Turbo DNA-free^TM^ Kit (Life Technologies, Grand Island, NY) to remove potential contaminating DNA that may lead to false-positive amplification. RNA quality and purity was confirmed with a Nanodrop spectrophotometer (Thermo Scientific). The resulting intact RNA was used for either RNAseq analysis or QPCR analyses (see below). RNAseq was performed using RNA from the bone samples, using methods as extensively described previously from our laboratory [7]. The only exception to this was that because ample total RNA was obtained from the bone biopsies, there was no need to amplify the mRNA species, as done previously [8].

Briefly, first strand cDNA was generated from ~100 ng of total RNA using DNA/RNA chimeric primers and reverse transcriptase, creating a cDNA/RNA hybrid, followed by second strand cDNA synthesis containing a DNA/RNA duplex. The resulting double-stranded cDNA products were modified by random priming and extension to create double-stranded products suitable for generating sequencing libraries. The double-stranded products then underwent blunt-end repair. Adapter molecules were ligated to the 5’ and 3’ ends of each fragment to facilitate PCR amplification of the fragments to produce the final library. Unique indices were created for each sample and incorporated at the adaptor ligation step for loading multiple samples per flow cell. Three distinct libraries were loaded per flow cell and sequenced on an Illumina HiSeq 2000 using TruSeq SBS sequencing software (version 3) and SCS data collection software (version 1.4.8). Base calling was performed using Illumina RTA (version 1.12.4.2). The RNAseq data associated with this manuscript are accessible through GEO Series accession number GSE72815.

### QPCR gene expression analyses

QPCR primers were designed using the Primer Express^®^ program (version 3.0.1, Applied Biosystems) and are available on request. QPCR reactions were run using the ABI Prism 7900HT Real time System (Applied Biosystems) with SYBR Green (Qiagen) as the detection method. Normalization for variations in input RNA was performed based on a panel of 10 housekeeping genes (*18S*, *G6PDH*, *GAPDH*, *GUSB*, *L13A*, *RPII*, *TBP*, *TUBA1B*, *Β2M*, *ACTB*) from which the 3 most stable housekeeping genes were selected using the geNorm algorithm (http://medgen.ugent.be/~jvdesomp/genorm/) [9,10], while the PCR Miner algorithm [11] was used to correct for variations in amplification efficiencies.

### Serum biochemical assays

Total 25(OH)D (within assay CV of 2.4% and between assay CV of 6.8%) was measured using liquid chromatography-tandem mass spectrometry (API 5000; Applied Biosystems-MDS Sciex, Foster City, CA). Creatinine was measured by isotope dilution mass spectroscopy; the estimated glomerular filtration rate (eGFR) was calculated using the Modification of Diet in Renal Disease (MDRD) equation [12]. Bone formation was assessed by serum amino-terminal propeptide of type I collagen (PINP; μg/L) as measured by double-antibody radioimmunoassay (within assay CV of 7.5%, between assay CV of 6.5%; DiaSorin, Stillwater, MN). Bone resorption was evaluated by serum cross-linked C-telopeptide of type I collagen (CTx; ng/mL) using an enzyme-linked immunosorbent assay (ELISA, within assay CV of 4.6%, between assay CV of 8.0%; Roche Diagnostics, Indianapolis, IN). Serum sclerostin levels were measured using two different techniques: 1) by ELISA (pg/mL, within- and between-assay CVs of <6%; Biomedica, Alpco, Salem, NH); and 2) using an electrochemiluminescense assay (pg/mL, within assay CV of 6%, between assay CV of 10%; Meso-Scale Discoveries [MSD] 96-Well MULTI-ARRAY Human Sclerostin Assay, Gaithersburg, MD), as previously described [13]. The MSD assay has a detection limit of 1 pg/mL (with a range of 1 to 10,000 pg/mL), and rigorous epitope mapping has demonstrated that this assay detects the full intact sclerostin molecule [14].

### Statistical analyses

Clinical characteristics and serum biochemistries were compared between groups using *t*-tests or ANOVA as appropriate. The QPCR analysis of the bone biopsies obtained from this cohort has been described in detail [6,7]. As previously described [8], the RNAseq data were analyzed using several steps: alignment, quality control, obtaining genomic features per sample and summarizing the data across samples for subsequent comparisons between samples (see below). Pair-end reads were aligned by TopHat 2.0.6 [15] against the hg19 genome build using the bowtie1 aligner option [16]. Initial assessment of quality control was made using RSeQC software [17] to estimate the distance between paired end reads, evaluate the sequencing depth for alternate splicing analysis, determine the rate of duplicate reads, evaluate coverage of reads across genes, etc. Gene counts were generated using HTseq software (http://www-huber.embl.de/users/anders/HTSeq/doc/overview.html) and gene annotation files were obtained from Illumina (http://cufflinks.cbcb.umd.edu/igenomes.html). Genes with median gene counts of <10 in both young and old groups were considered “non-expressed”, as previously described [8]. Conditional quantile normalization using the cqn Bioconductor package [18] was applied to the RNAseq data to reduce variability introduced by GC content, gene size, and total gene counts per sample. Significance in differential expression was determined using the edgeR Bioconductor package [19] assuming a negative binomial error structure accounting for the cqn derived offset. We used a correction for the false discovery rate (FDR, *q*-value) in order to account for multiple comparisons, specifying a *q*-value of < 0.10, as is generally accepted [16,17].

Pathway enrichment analysis was conducted using the Ingenuity Pathway Analysis software (IPA, Ingenuity Systems, Inc., Redwood City, CA, USA; http://www.ingenuity.com) to identify significant canonical pathways in which the Differentially Expressed Genes (DEGs) in the tested samples were enriched. The IPA program applies Fisher’s exact test to calculate a *p*-value that represents the probability of the DEGs in the pathway being found together due to random chance. The Benjamini-Hochberg FDR (*q* < 0.10) correction was also applied in order to account for multiple comparisons in the IPA. In addition, IPA was used to determine networks associated with aging in the tested samples based on DEGs. For network generation, a dataset with gene identifiers and corresponding fold change values was uploaded into IPA. Default settings were used to identify differentially regulated molecules. These molecules were overlaid onto a global molecular network (contained in the Ingenuity Knowledge Base) and algorithmically connected. The functional analysis in IPA determined the biological functions and diseases associated with each network.

The transcription factor binding site analysis was conducted using the C3 module (motif gene sets) from the Molecular Signatures Database (MSigDB; http://www.broadinstitute.org/gsea/msigdb/collections.jsp#C3) [20]. This analysis identifies common cis-regulatory DNA motifs that are statistically enriched in the query gene list. The transcription factor binding sites are defined in the TRANSFAC (version 7.4; http://www.gene-regulation.com) database. These motifs are catalogued and represent known or likely DNA regulatory elements contained within the DNA sequence surrounding the transcriptional start site of a particular gene of interest [21]. Upstream regulator analysis, a component of the IPA software package, was performed as described in the IPA software documentation.

## Results

### Descriptive characteristics

Clinical characteristics of the young, old, and E groups are shown in Table 1. In this cohort of healthy women, the premenopausal women were, as expected, much younger (mean age ± SD, 30.3 ± 5.4 years) than both groups (old and E therapy) of postmenopausal women. By contrast, the old (age 73.1 ± 6.6 years) and E (age 70.5 ± 5.2 years) groups did not differ in age. Whereas the young women were taller than both groups of old women, the old and E groups were similar in height. In addition, none of the groups differed in weight or BMI. All women had sufficient levels of total 25(OH)D and creatinine, levels of which were in the normal range and did not differ among the groups. By contrast, eGFR was significantly higher in young as compared to both groups of old women. At baseline, the young women tended to have lower serum levels of CTx (a marker of bone resorption), but similar serum levels of PINP (a marker of bone formation), as compared to both groups of old women. Further, as compared to the old non-treated women, PINP and CTx levels were not different at baseline in the old E-treated women. As previously described [7], short-term E therapy markedly increased serum PINP (by 55.4%, *p* < 0.001), reflecting the effects of E in enhancing bone formation; this increase is followed by a subsequent decrease in PINP levels [7] due to the coupling of bone formation and bone resorption, which is uniformly reduced by E therapy. The latter is reflected by the decrease in serum CTx (by 28.6%, *p* < 0.001) levels relative to baseline after 3 weeks of E treatment (Table 1). By contrast, neither parameter changed in the non-treated old women over the same time course.

**Table 1:** Clinical characteristics and biochemical markers in the young, old, and estrogen (E)-treated women.

	Young	Old	E
n	19	19	20
Age (years)	30.3 ± 5.4^a^	73.1 ± 6.6	70.5 ± 5.2^c^
Height (cm)	167 ± 6^a^	163 ± 7	162 ± 4^c^
Weight (kg)	72.3 ± 14.1	72.1 ± 11.7	75.9 ± 15.5
BMI (kg/m^2^)	26.2 ± 5.3	27.3 ± 3.7	28.7 ± 4.8
eGFR, ml/min/1.73m^2^	83.5 ± 18.9^a^	70.1 ± 7.6	71.6 ± 14.6^c^
*Serum Biochemistries*			
Total 25(OH)D (ng/ml)	36.3 ± 13.3	37.4 ± 7.7	40.9 ± 14.4
Creatinine (mg/dl)	0.84 ± 0.15	0.81 ± 0.08	0.82 ± 0.15
PINP			
Baseline (μg/l)	42.8 ± 16.2	50.6 ± 17.0	42.0 ± 20.8
3 weeks (μg/l)	NA	52.4 ± 23.4	62.6 ± 20.4
Percent change (%)	NA	2.1 ± 22.4^b^	55.4 ± 27.4
CTx			
Baseline (ng/ml)	0.32 ± 0.13^a^	0.53 ± 0.25	0.44 ± 0.17
3 weeks (ng/ml)	NA	0.52 ± 0.25^b^	0.32 ± 0.20
Percent change (%)	NA	-1.7 ± 14.8^b^	-28.6 ± 19.1

Values are presented as means ± SD. E = estrogen; BMI = body mass index; 25(OH)D = 25-hydroxyvitamin D; eGFR = estimated glomerular filtration rate; PINP = amino-terminal propeptide of type I collagen; CTx = cross-linked C-telopeptide of type I collagen; NA = not applicable.

^a^ *p* < 0.05 Young versus Old;

^b^ *p* < 0.05 Old versus E;

^c^ *p* < 0.05 Young versus E.

### High-throughput RNAseq of bone biopsies

We next extended our previous QPCR analyses of the bone biopsies [6,7] to include identification of genes and pathways altered with aging and in response to E therapy using high-throughput RNAseq to compare the skeletal transcriptome of the young versus old and old versus E-treated women. Using fairly stringent criteria (median count ≥ 10, FDR [*q*] < 0.10), 678 genes were altered with aging (in old relative to young subjects), including 446 up- and 232 down-regulated genes, respectively (S1 Table). Additional adjustment for height and eGFR, two variables that differed between the young and old subjects, resulted in essentially no change in the findings (data not shown). By contrast, using the same criteria, only 21 genes were altered by E therapy (in E-treated relative to non-treated old subjects), including 18 up- and 3 down-regulated genes, respectively (S2 Table).

### QPCR confirmation of RNAseq results

Among the genes in S1 and S2 Tables, a number have been previously shown to be altered in human bone biopsies with aging [6] or E therapy [7] using QPCR, implying that our RNAseq analysis was accurate and reliable. In order to validate this further, we examined the association of gene expression levels between RNAseq and QPCR in a randomly selected subset of genes significantly altered with aging (*q* < 0.10 by RNAseq, n = 46). As evident in S1 Fig, a very strong correlation (*r* = 0.95, *p* < 0.001) was observed for gene expression fold changes between RNAseq and QPCR.

### Ingenuity Pathway Analysis (IPA)

In order to interrogate the specific canonical cellular pathways altered with skeletal aging, we performed IPA on the young versus old RNAseq dataset. It should be noted, however, that IPA was not run on the old versus E treatment dataset due to the comparatively fewer genes altered in response to E therapy. The IPA analysis identified 73 canonical pathways that were altered with aging at the *p* < 0.05 level (S3 Table). After applying a *q* < 0.10, a total of 12 pathways remained significant (Table 2). Although a number of pathways that were altered with aging at the *p* < 0.05 level did not meet FDR criteria (*q* < 0.10), several of these pathways have known functions in bone metabolism and therefore warrant consideration as potential targets to treat osteoporosis; Table 3 lists 10 cellular pathways that fell in this category.

**Table 2:** Pathways altered (*q* < 0.10) in the young versus old RNAseq dataset based on pathway analysis using the Ingenuity Pathway Analysis software (see *Statistical analyses*). The associated *p*-value, false discovery rate (*q*), and ratio for each pathway, as well as each gene fold change (in parenthesis) are provided.

*Pathway*	*p*	*q*	*Ratio*	*Genes*
Hepatic Fibrosis / Hepatic Stellate Cell Activation	0.000	0.0003	0.106	*COL4A1*(1.39), *LEP*(2.11), *COL6A2(1*.*31)*, *FGF2(1*.*51)*, *COL2A1(0*.*11)*, *FGFR2(1*.*33)*, *COL4A2(1*.*34)*, *COL15A1(0*.*11)*, *COL28A1(1*.*55)*, *PGF(1*.*65)*, *FGF1(1*.*61)*, *MET(1*.*78)*, *COL5A3(1*.*34)*, *COL6A1(1*.*43)*, *CCL2(1*.*80)*, *PDGFRA(1*.*36)*, *CCL21(2*.*77)*, *A2M(1*.*27)*, *AGTR1(1*.*49)*, *COL7A1(1*.*42)*, *PDGFRB(1*.*39)*
Notch Signaling	0.000	0.021	0.184	*NOTCH4(1*.*26)*, *NOTCH3(1*.*41)*, *JAG2(1*.*38)*, *HES1(1*.*37)*, *DLL4(1*.*36)*, *HEY1(1*.*59)*
Regulation of the Epithelial-Mesenchymal Transition Pathway	0.000	0.021	0.087	*NOTCH3(1*.*41)*, *JAG2(1*.*38)*, *SNAI2(1*.*39)*, *FGF2(1*.*51)*, *FGFR2(1*.*33)*, *PARD6A(0*.*80)*, *FGF1(1*.*61)*, *MET(1*.*78)*, *PIK3R3(1*.*25)*, *NOTCH4(1*.*26)*, *FZD4(1*.*31)*, *PIK3CG(0*.*82)*, *MRAS(1*.*26)*, *LEF1(0*.*76)*, *FGF7(1*.*53)*, *PDGFRB(1*.*39)*
Coagulation System	0.001	0.058	0.17	*F8(1*.*31)*, *PROC(0*.*72)*, *VWF(1*.*43)*, *TFPI(1*.*23)*, *A2M(1*.*27)*, *PLAT(1*.*91)*
Inhibition of Matrix Metalloproteases	0.001	0.062	0.15	*HSPG2(1*.*54)*, *TIMP3(1*.*60)*, *TIMP4(1*.*56)*, *MMP25(0*.*79)*, *A2M(1*.*27)*, *MMP19(1*.*25)*
Bladder Cancer Signaling	0.001	0.062	0.10	*FGF2(1*.*51)*, *CDKN1A(1*.*31)*, *MRAS(1*.*26)*, *MMP25(0*.*79)*, *CCND1(1*.*31)*, *FGF7(1*.*53)*, *PGF(1*.*65)*, *FGF1(1*.*61)*, *MMP19(1*.*25)*
eNOS Signaling	0.001	0.062	0.08	*PIK3R3(1*.*25)*, *AQP7(1*.*74)*, *LPAR4(0*.*67)*, *CAMK4(0*.*65)*, *CNGA4(0*.*51)*, *ADCY5(1*.*50)*, *PIK3CG(0*.*82)*, *GUCY1A2(0*.*64)*, *CAV1(1*.*64)*, *AQP1(1*.*19)*, *NOS3(1*.*31)*, *PGF(1*.*65)*
HGF Signaling	0.001	0.062	0.10	*MET(1*.*78)*, *PIK3R3(1*.*25)*, *DOCK1(1*.*22)*, *MAP3K6(1*.*29)*, *PIK3CG(0*.*82)*, *CDKN1A(1*.*31)*, *MAP3K1(0*.*88)*, *MRAS(1*.*26)*, *CCND1(1*.*31)*, *MAP3K2(0*.*89)*
Atherosclerosis Signaling	0.001	0.062	0.09	*IL33(1*.*34)*, *PLA2G16(1*.*30)*, *COL5A3(1*.*34)*, *ALOX15B(0*.*71)*, *PLA2G2D(0*.*66)*, *CCL2(1*.*80)*, *CMA1(4*.*18)*, *CD36(1*.*18)*, *PLA2R1(1*.*37)*, *COL2A1(0*.*11)*, *TPSAB1(2*.*92)*, *TPSB2(2*.*12)*
Glioma Invasiveness Signaling	0.002	0.065	0.12	*PIK3R3(1*.*25)*, *TIMP3(1*.*60)*, *TIMP4(1*.*56)*, *RHOC(1*.*16)*, *PIK3CG(0*.*82)*, *MRAS(1*.*26)*, *RHOJ(1*.*31)*
Acute Phase Response Signaling	0.002	0.071	0.08	*C1R(1*.*37)*, *PIK3R3(1*.*25)*, *IL33(1*.*34)*, *SERPING1(1*.*30)*, *F8(1*.*31)*, *PIK3CG(0*.*82)*, *C1S(1*.*53)*, *MAP3K1(0*.*88)*, *MRAS(1*.*26)*, *VWF(1*.*43)*, *OSMR(1*.*43)*, *CP(1*.*22)*, *A2M(1*.*27)*
Antiproliferative Role of Somatostatin Receptor 2	0.003	0.095	0.11	*PIK3R3(1*.*25)*, *GUCY2D(0*.*71)*, *PIK3CG(0*.*82)*, *CDKN1A(1*.*31)*, *MRAS(1*.*26)*, *GUCY1A2(0*.*64)*, *GNG7(0*.*83)*

**Table 3:** Selected pathways of interest with known function in bone in the young versus old RNAseq dataset based on pathway analysis using the Ingenuity Pathway Analysis software (see *Statistical analyses*). The associated *p*-value, false discovery rate (*q*), and ratio for each pathway, as well as each gene fold change (in parenthesis) are provided.

*Pathway*	*p*	*q*	*Ratio*	*Genes*
Notch Signaling	<0.001	0.021	0.19	*NOTCH4(1*.*26)*, *NOTCH3(1*.*41)*, *JAG2(1*.*38)*, *HES1(1*.*37)*, *DLL4(1*.*36)*, *HEY1(1*.*59)*
Inhibition of Matrix Metalloproteases	0.002	0.062	0.16	*HSPG2(1*.*53)*, *TIMP3(1*.*60)*, *TIMP4(1*.*56)*, *MMP25(0*.*79)*, *A2M(1*.*27)*, *MMP19(1*.*25)*
eNOS Signaling	0.002	0.062	0.09	*PIK3R3(1*.*25)*, *AQP7(1*.*74)*, *LPAR4(0*.*67)*, *CAMK4(0*.*65)*, *CNGA4(0*.*51)*, *ADCY5(1*.*50)*, *PIK3CG(0*.*82)*, *GUCY1A2(0*.*64)*, *CAV1(1*.*64)*, *AQP1(1*.*19)*, *NOS3(1*.*31)*, *PGF(1*.*65)*
Nitric Oxide Signaling in the Cardiovascular System	0.005	0.102	0.09	*PIK3R3(1*.*25)*, *CAMK4(0*.*65)*, *GUCY2D(0*.*71)*, *PIK3CG(0*.*82)*, *GUCY1A2(0*.*64)*, *CAV1(1*.*64)*, *NOS3(1*.*31)*, *PGF(1*.*65)*, *PDE1C(1*.*60)*
PDGF Signaling	0.015	0.124	0.09	*PIK3R3(1*.*25)*, *PIK3CG(0*.*82)*, *MRAS(1*.*25)*, *PDGFRA(1*.*36)*, *CAV1(1*.*64)*, *PDGFRB(1*.*39)*
PTEN Signaling	0.019	0.130	0.08	*PIK3R3(1*.*25)*, *PIK3CG(0*.*82)*, *CDKN1A(1*.*31)*, *MRAS(1*.*25)*, *PDGFRA(1*.*36)*, *FGFR2(1*.*33)*, *MAGI2(1*.*36)*, *CCND1(1*.*31)*, *PDGFRB(1*.*39)*
Gαi Signaling	0.020	0.132	0.08	*ADRA2B(1*.*61)*, *GPR17(1*.*74)*, *APLNR(1*.*61)*, *NPY1R(1*.*95)*, *ADCY5(1*.*50)*, *MRAS(1*.*26)*, *CAV1(1*.*64)*, *GNG7(0*.*83)*, *AGTR1(1*.*49)*
Wnt/β-catenin Signaling	0.026	0.156	0.07	*SOX17(1*.*58)*, *SOX4(0*.*84)*, *SOX7(1*.*33)*, *FZD4(1*.*31)*, *CDH5(1*.*35)*, *SFRP5(0*.*42)*, *LEF1(0*.*76)*, *SFRP1(1*.*46)*, *SOX18(1*.*60)*, *SOX13(1*.*45)*, *CCND1(1*.*31)*
Oncostatin M Signaling	0.027	0.188	0.12	*TIMP3(1*.*60)*, *EPAS1(1*.*34)*, *MRAS(1*.*26)*, *OSMR(1*.*43)*
HIF1α Signaling	0.021	0.236	0.08	*PIK3R3(1*.*25)*, *PIK3CG(0*.*82)*, *MRAS(1*.*26)*, *CAV1(1*.*64)*, *NOS3(1*.*31)*, *MMP25(0*.*79)*, *PGF(1*.*65)*, *MMP19(1*.*25)*

**Fig 2:**
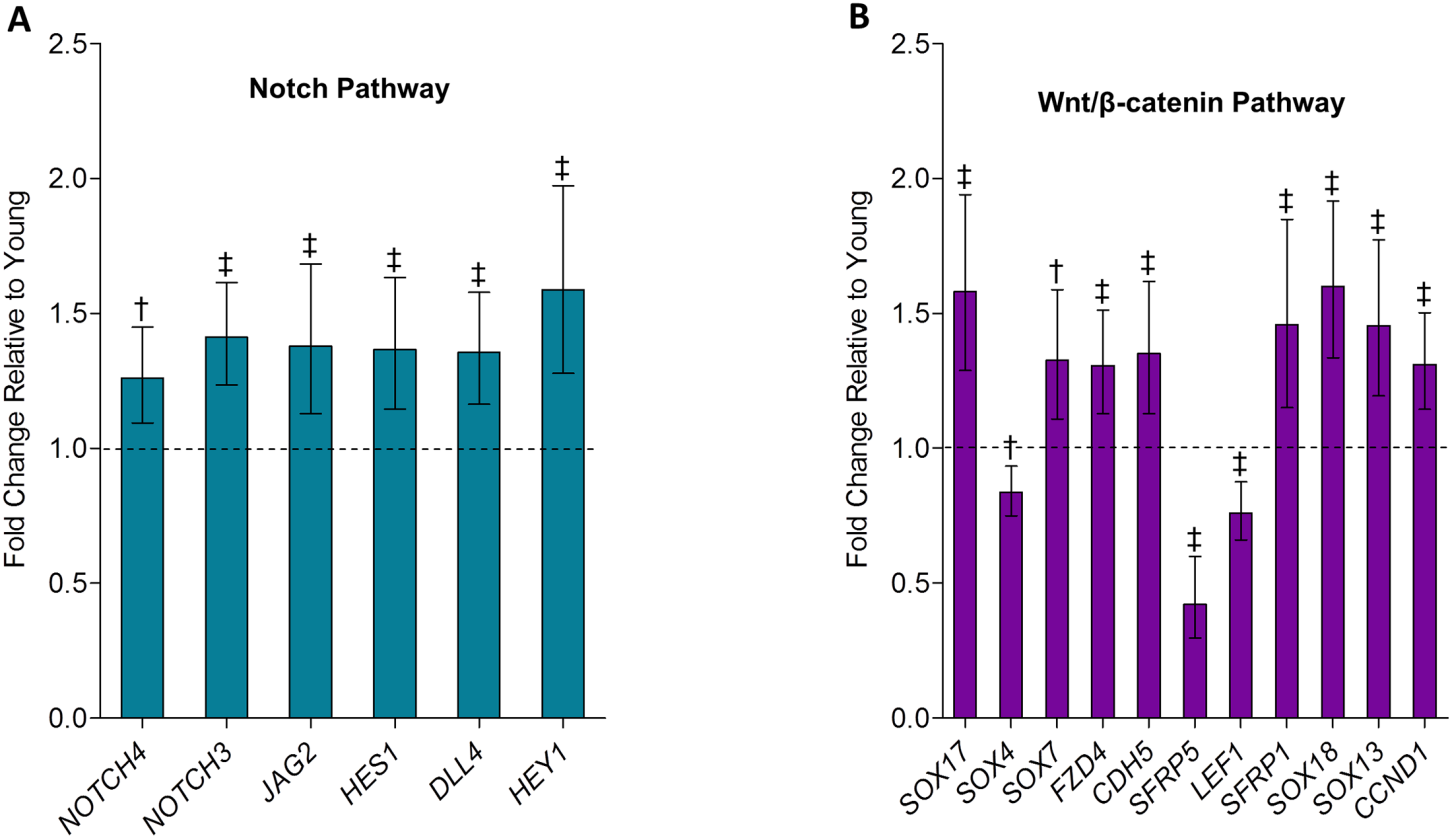
(A) Notch pathway and (B) Wnt/β-catenin pathway genes are altered with aging in human bone biopsies. Genes in the Notch pathway and Wnt/β-catenin pathway are significantly altered in old relative to young women revealed by RNAseq based on pathway analysis using the Ingenuity Pathway Analysis software (see *Statistical analyses*). Values are presented as median fold changes (95% CIs) for old relative to young subjects. ^†^ *p* < 0.01; ^‡^ *p* < 0.001.

Particularly noteworthy, and consistent with our previous study using QPCR [6], was the significant alteration (*q* = 0.021) of the Notch signaling pathway with aging in the bone biopsies (Table 2). Indeed, expression levels of key regulatory genes from the Notch pathway including *NOTCH3* and *NOTCH4* (both single-pass transmembrane cell surface receptors), as well as *JAG2* and *DLL4* (two Notch receptor ligands that cause proteolytic cleavage of Notch receptors upon binding) and two critical direct downstream Notch pathway targets (*HES1* and *HEY1*) were significantly up-regulated in bone biopsies of old versus young subjects (Fig 2A). It is also noteworthy that the Wnt/β-catenin signaling pathway was altered with aging, although as noted above, this pathway fell into the category of bone-related pathways with *p* < 0.05, but *q* > 0.10 (Table 3). In this pathway, transcriptional levels of the genes for *SOX4*, *SFRP5*, and *LEF1* were decreased, whereas levels for *SOX17*, *SOX7*, *FZD4*, *CDH3*, *SFRP1*, *SOX18*, *SOX13*, and *CCND1* were all increased in bone biopsies of old versus young women (Fig 2B). By contrast, however, there were no changes in any of the lipoprotein receptor-related proteins previously linked to bone (e.g., LRP4, LRP5, and LRP6) or the associated DKK family of Wnt antagonists with aging. Finally, as evident in Table 3, additional pathways with known roles in bone metabolism, including Inhibition of Matrix Metalloproteases, eNOS signaling, Nitric Oxide Signaling in the Cardiovascular System, PDGF Signaling, Gαi Signaling, and Oncostatin M Signaling were all altered in bone biopsies of old versus young subjects (at least at the *p* < 0.05 level, but only the first 2 at the *q* < 0.10 level).

### Network analyses

**Fig 3:**
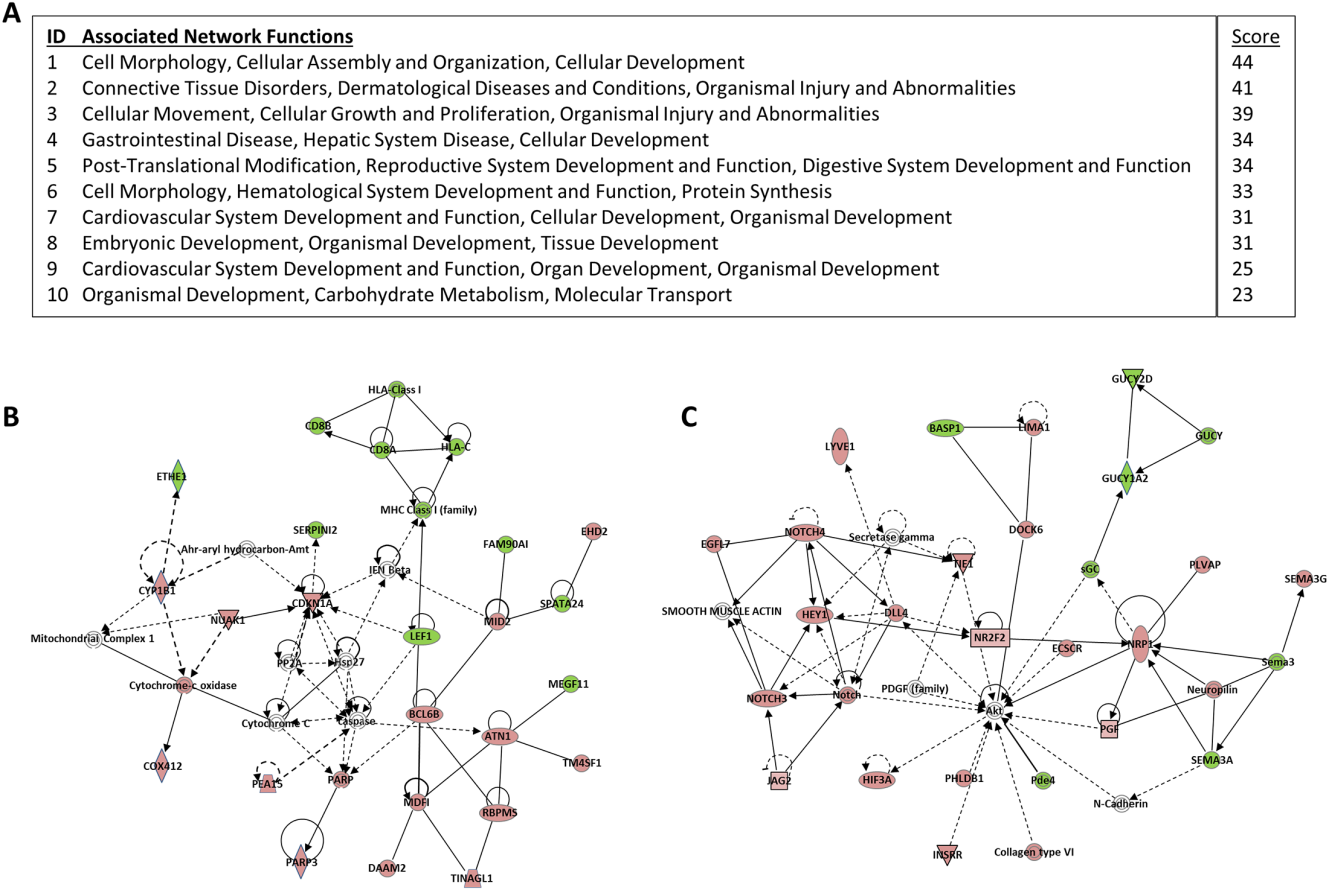
Networks derived from age-related differentially expressed genes (DEGs) in bone. (A) Top 10 scoring networks and associated network functions derived from the 678 DEGs altered (median count ≥ 10, *q* < 0.10) in the young versus old dataset determined using the Ingenuity Pathway Analysis software (see *Statistical Analysis*). (B) Network of cell morphology, hematological system development and function, and protein synthesis (score = 33); key molecules implicated in bone metabolism include *LEF1* and *CDKN1A* (p21). (C) Network of cardiovascular system development and function, cellular development, and organismal development (score = 31); key molecules implicated in bone metabolism include Notch signaling components (*NOTCH3*, *NOTCH4*, *HEY1*, *JAG2*, and *DLL4*) and *SEMA3A*. Green indicates downregulated genes, and red indicates upregulated genes.

We next utilized IPA to identify networks based on the 678 network-eligible molecules that were altered (median count ≥ 10, *q* < 0.10) in bone with aging S4 Table lists all identified networks, as well as their associated scores and network molecules). The top 10 highest scored networks are listed in Fig 3A. Particularly noteworthy, genes identified among the highest scored networks (Fig 3B) included *LEF1*, which plays an important role in Wnt/β-catenin signaling [22,23], as well as *CDKN1A* (p21), a key mediator of cellular senescence [24]. Other molecules implicated in bone metabolism identified among the highest scored networks were Notch signaling components (*NOTCH3*, *NOTCH4*, *HEY1*, *JAG2*, and *DLL4*) (Fig 3C) and *SEMA3A*; the latter regulates bone metabolism via sensory innervations [25].

### Identification of enriched cis-regulatory DNA motifs

In addition to the IPA-derived canonical pathways associated with the DEGs from the young versus old RNAseq dataset, it is also of interest to identify common cis-regulatory DNA elements that are enriched with aging, which then can provide clues regarding shared molecular mechanisms involved in the regulation of these DEGs. Therefore, in order to identify putative cis-regulatory elements that are enriched in the promoter regions of the 678 DEGs from the young versus old RNAseq dataset, we queried the C3 module (motif gene sets) from the Molecular Signatures Database [MSigDB; [20]]. This analysis identified statistically enriched cis-regulatory DNA sequences contained within 2-kilobases (kb) surrounding the transcription start site for each gene (e.g. from -2kb to +2kb). Table 4 summarizes the top 20 cis-regulatory DNA motifs based on *p*-value (a complete list of the top 100 motifs can be found in S5 Table). Particularly noteworthy was the identification of two related DNA motifs for the *LEF1* transcription factor, a classical downstream target of the Wnt/β-catenin signaling pathway [22,23]. This is consistent with our IPA and QPCR analyses (Table 3 and Fig 2B, respectively) and suggests that aging is associated with alterations in Wnt signaling in bone. The specific DEGs from the young versus old RNAseq dataset containing each of the *LEF1* cis-regulatory motifs are shown in S6 Table.

**Table 4:** Top 20 transcription factor binding sites enriched in the DEGs from the young versus old RNAseq dataset. The ratio (the percentage of genes in the overlap between the 678 DEGs and the MSigDB gene set), *P*-value and false discovery rate (FDR) are provided.

*Symbol*	*Transcription Factor*	*DNA Motif*	*Ratio*	*p-value*	*q*
MAZ	MYC-associated zinc finger protein	GGGAGGRR	0.0487	1.39E-30	8.57E-28
TCF3	transcription factor 3	CAGGTG	0.0462	1.01E-29	3.10E-27
Unknown		AACTTT	0.0518	5.52E-29	1.13E-26
JUN	jun proto-oncogene	TGANTCA	0.0632	5.00E-26	7.68E-24
LEF1	lymphoid enhancer-binding factor 1	CTTTGT	0.0469	1.99E-24	2.45E-22
FOXO4	forkhead box O4	TTGTTT	0.0445	1.35E-22	1.38E-20
REPIN1	replication initiator 1	CAGCTG	0.0505	3.87E-22	3.10E-20
NFATC1	nuclear factor of activated T-cells	TGGAAA	0.0457	4.04E-22	3.10E-20
MEIS1	Meis homeobox 1	TGACAGNY	0.0626	4.10E-19	2.80E-17
PAX4	paired box 4	GGGTGGRR	0.0478	7.12E-17	4.38E-15
LEF1	lymphoid enhancer-binding factor 1	CTTTGA	0.0487	1.07E-16	5.99E-15
ETS2	v-ets avian erythroblastosis virus E26 oncogene homolog 2	RYTTCCTG	0.0505	4.73E-16	2.42E-14
Unknown		WTTGKCTG	0.0696	5.33E-15	2.52E-13
TBP	TATA box binding protein	TATAAA	0.0446	1.49E-14	6.53E-13
FOXA1	forkhead box A1	TGTTTGY	0.0568	3.11E-14	1.27E-12
Unknown		TTANTCA	0.0503	4.18E-14	1.61E-12
TCF8	transcription factor 8	CAGGTA	0.0542	7.90E-14	2.86E-12
Unknown		CTTTAAR	0.0493	8.95E-14	3.06E-12
MYOD1	myogenic differentiation 1	GCANCTGNY	0.0497	2.18E-13	6.74E-12
Unknown		WTGAAAT	0.0600	2.19E-13	6.74E-12

### Upstream Regulator Analytic (URA) tool

Another interesting feature of the IPA software package is the Upstream Regulator Analytic (URA) function. This tool enables identification of putative upstream regulators based on the DEGs identified in the canonical pathway analysis, and predicts whether these regulators are activated or inhibited based on the directionality of the DEGs. The URA tool reports an Activation *z*-score, where *z*-scores ≥2 are considered activated and *z*-scores ≤-2 are considered inhibited. Table 5 summarizes the top 10 activated and inhibited upstream regulators in the young versus old RNAseq dataset. The top activated upstream regulator was the growth factor TGFβ1. Interestingly, the top repressed upstream regulator was SMAD7, which is an inhibitory SMAD involved in ression of TGFβ signaling [26]. A complete list of upstream regulators with *z*-scores ≥2 or ≤-2 is provided in S7 Table.

**Table 5:** Top 10 activated and repressed upstream regulators in the young versus old dataset.

*Upstream Regulator*	*Category*	*z score* ^*2*^	*p-value of overlap* ^*3*^
*Activated*			
TGFB1	growth factor	4.868	1.58E-12
Cg	complex	4.679	9.84E-10
Vegf	group	4.444	1.33E-09
RAF1	kinase	3.495	2.95E-04
HGF	growth factor	3.338	1.23E-06
IL17A	cytokine	3.229	2.52E-03
VEGFA	growth factor	3.224	1.49E-05
AGT	growth factor	3.205	6.51E-09
Tgf beta	group	3.190	7.20E-05
SP1	transcription regulator	3.140	2.72E-03
*Inhibited*			
SMAD7	transcription regulator	-3.043	4.54E-05
GMNN	transcription regulator	-3.000	6.21E-05
SOX3	transcription regulator	-2.828	1.50E-03
SOX1	transcription regulator	-2.828	1.95E-04
Alpha catenin	group	-2.765	5.17E-04
COL18A1	other	-2.745	3.47E-03
ERBB3	kinase	-2.63	8.06E-03
HAND1	transcription regulator	-2.433	5.26E-05
*miR-124-3p*	mature microRNA	-2.423	1.11E-02
N-cor	group	-2.401	3.52E-04

### Bone and serum sclerostin levels

**Fig 4:**
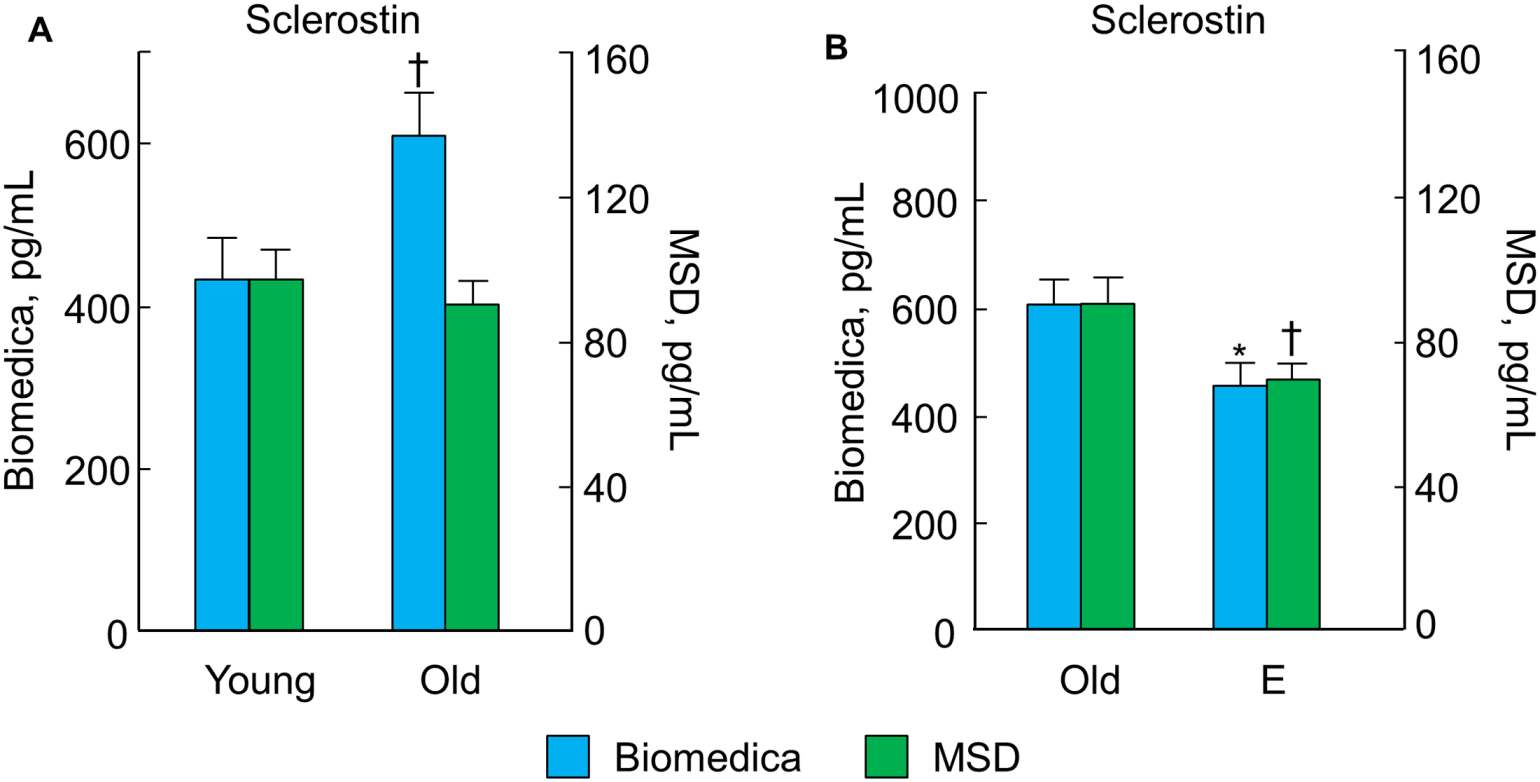
Effects of age and estrogen (E) on serum sclerostin levels. Serum sclerostin levels in the young versus old subjects (A) and in the old versus E-treated subjects (B) by the Biomedica and Meso Scale Discovery (MSD) assays. Data are mean ± SEM; note the difference in scales for the two sclerostin assays. *p < 0.05; ^†^ *p* < 0.01.

Since we have previously reported age [6] and E [7] effects on sclerostin (*SOST*) mRNA levels by QPCR in these women, we next examined *SOST* gene expression levels in the RNAseq dataset, as well as serum levels of sclerostin in the young, old, and E-treated subjects using two different sclerostin assays. Consistent with our previous QPCR data [6], [7], there was no effect of age on *SOST* mRNA levels by RNAseq (fold change in the old versus young = 0.99, *p* = 0.981), whereas E treatment did significantly reduce *SOST* mRNA levels (fold change in the E-treated versus untreated old women = 0.59, *p* = 0.026), although this change did not meet FDR criteria (*q* = 0.46). Interestingly, although serum sclerostin levels were significantly higher in the old versus young subjects using the Biomedica assay, they were no different in the old versus young subjects using the MSD assay (Fig 4A). By contrast, consistent with both the QPCR and RNAseq data, serum sclerostin levels using both assays were reduced in old women by E therapy (Fig 4B). Thus, the MSD assay more accurately reflected changes in bone *SOST* mRNA levels with aging and E treatment, showing concordant changes in circulating sclerostin levels under both conditions.

### Genes altered by E therapy

**Fig 5:**
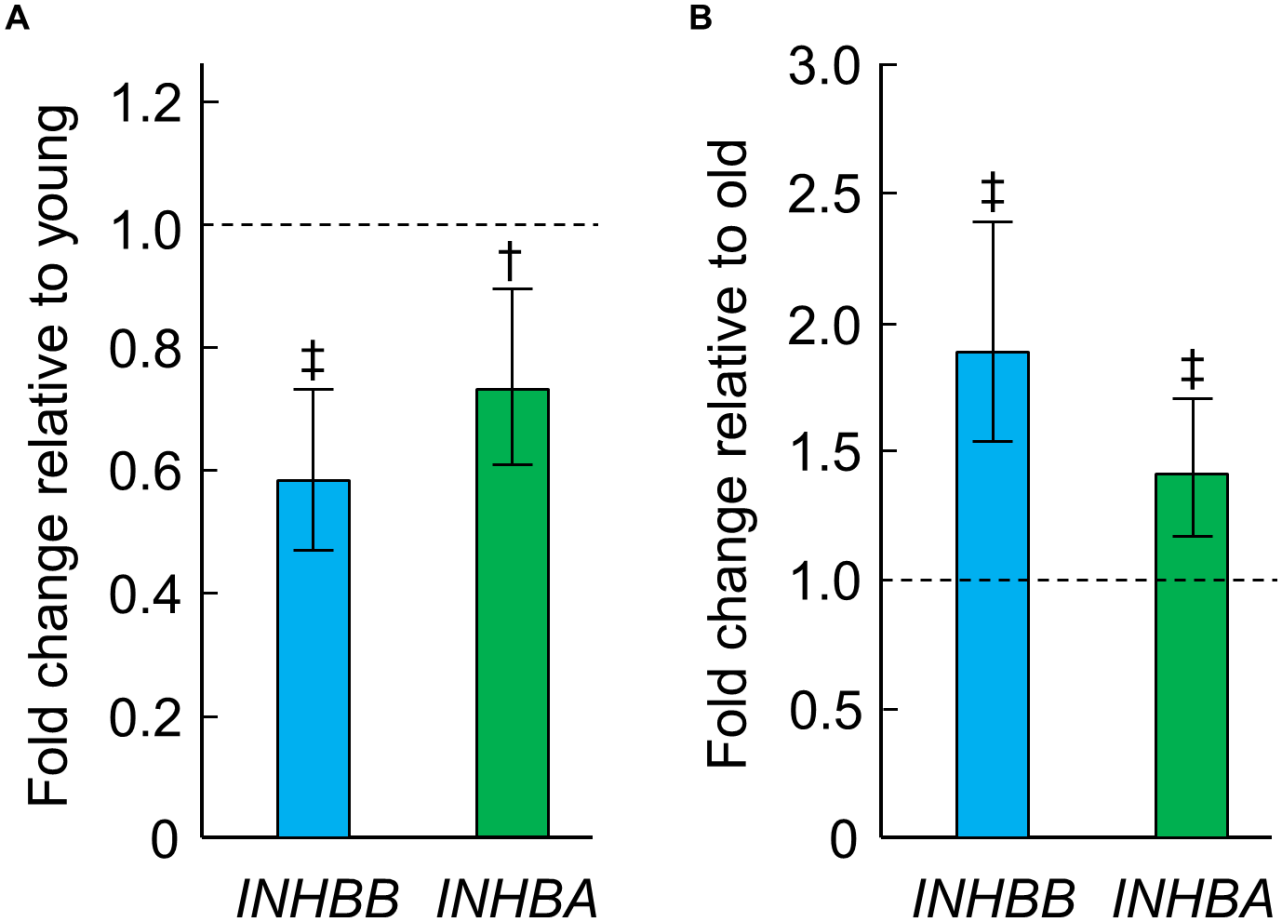
Effects of age and estrogen (E) on gene expression of *INHBA* and *INHBB*. Bone *INHBB* and *INHBA* gene expression levels by RNAseq in the old relative to young women (A) and in the E-treated relative to untreated old women (B). Data are shown as median fold changes (95% CIs). ^†^ *p* < 0.01; ^‡^ *p* < 0.001.

Finally, we focused on the 21 genes in bone tissue altered in old women in response to E therapy as revealed by RNAseq (S2 Table). Particularly noteworthy were the marked increases in expression of *LGR5* (by 3.9-fold) and *PPARGC1A* (by 3.7-fold), while the expression of *C19orf80* (coding for Betatrophin) decreased (by 0.5-fold) in response to E therapy. Furthermore, the expression of *INHBB* (inhibin, beta B), which significantly decreased with aging (Fig 5A; *q* < 0.001), was restored to young adult levels (Fig 5B; *q* < 0.001) in response to E therapy. Correspondingly, expression of *INHBA* (inhibin, beta A) followed the same pattern with aging (Fig 5A; *p* < 0.01; *q* = 0.118) and in response to E therapy (Fig 5B; *p* < 0.001; *q* = 0.179), although these gene expression changes did not meet FDR criteria (*q* < 0.10).

## Discussion

It is important to more precisely define the specific genes and pathways in human bone that are altered with aging and E deficiency for several reasons. First, direct identification of the underlying mechanisms for impaired bone formation with aging in humans is a significant gap in knowledge. Second, such studies may lead to the development of new skeletal biomarkers, which could result in the identification of circulation-based biomarkers to define patients most at risk for bone loss. Third, because virtually all current skeletal therapies target osteoclast-mediated bone resorption, the results of such studies may lead to novel approaches to stimulate bone formation in patients and prevent or reverse age-related bone loss. However, what has been lacking is an unbiased assessment of the potential genes and pathways regulated by aging and E in human bone.

Based on these considerations, the genes identified in this report using RNAseq that were altered in human bone with aging and in response to short-term E therapy may represent novel skeletal biomarkers. Furthermore, our results demonstrate that aging significantly altered a total of 12 canonical pathways in bone tissue, including a subset known to regulate bone metabolism (e.g., Notch). Interestingly, a number of the identified pathways and genes were previously unrecognized as changing in bone with aging. Perhaps unexpectedly, many of these pathways and genes have critical functions in other tissues and disease processes, although their role(s) in the pathogenesis of skeletal fragility requires additional investigation.

Consistent with our previous report using QPCR [6], our RNAseq analysis revealed that genes associated with Notch signaling were significantly altered in bone with aging. Based on transgenic mouse models, some genetic mutations in individual Notch surface receptors or certain Notch receptor ligands can be embryonically lethal [27,28,29], whereas others have no apparent skeletal effects [30,31,32,33,34]. More recently, Notch signaling has been shown to impair osteoblast maturation and differentiation through both HES and HEY family members, which inhibit Runx2 transcriptional activity through direct physical interactions, as demonstrated in various *in vitro* culture systems (e.g., MC3T3 cells [35], COS7 cells [36], and both CHO and ST2 cells [37]). Moreover, mutant mice overexpressing HEY1 develop an osteopenic phenotype [38]. Thus, given the crucial functions of Notch signaling in osteoblast maturation and differentiation, our finding that the Notch signaling pathway was altered in human bone with aging and specifically, that levels of both *HES1* and *HEY1* were significantly up-regulated in bone of old relative to young subjects, indicate that increased Notch signaling may contribute to impaired bone formation with aging. Moreover, these findings suggest that bone formation *in vivo* may be stimulated by targeted ression of the Notch signaling pathway [37].

Although originally discovered in rare human mutations affecting bone [39,40,41,42,43,44], mouse genetics has confirmed the importance of canonical Wnt signaling in the regulation of bone homeostasis [45]. Overall, activation of this pathway leads to increased bone formation, whereas inhibition leads to reduced bone formation. Thus, components of the Wnt pathway are currently targets of therapeutic interventions to restore bone mass and strength in aging humans. Perhaps surprisingly, we did not find that any of the genes encoding for the 19 known mammalian Wnt proteins were altered in human bone with aging or in response to E therapy. Similarly, none of the lipoprotein receptor-related proteins previously linked to bone (e.g., LRP4, LRP5, and LRP6) or the associated DKK family of Wnt antagonists were regulated in aging bone. Interestingly, however, another antagonist of Wnt signaling, *SFRP1*, was significantly up-regulated in bone of old relative young women. Capable of forming a complex with Frizzled receptors to thereby inhibit Wnt signaling [46], *SFRP1* up-regulation may play a causal role in ressing bone formation with aging. Importantly, *LEF1* mRNA levels were significantly reduced with aging, and *LEF1* was found among the highest scored IPA networks (Fig 3B). In addition, two related DNA motifs for the *LEF1* transcription factor were identified among the top cis-regulatory DNA elements enriched with aging. Since *LEF1* is a classical Wnt target gene [22,23,47], these findings are consistent with reduced Wnt action in bone with aging, perhaps related to increased *SFRP1* expression.

Mounting data in both animals and humans suggest that inhibitors of sclerostin (encoded by *SOST*), an osteocyte-secreted soluble Wnt/β-catenin signaling pathway antagonist, can stimulate bone formation. Using the Biomedica assay, previous studies in humans have demonstrated that serum levels of sclerostin increase with advancing age [6,48]. However, a concern with the Biomedica sclerostin assay is that it uses an anti-sclerostin antibody that may detect differential forms or proteolytic fragments of sclerostin [49]. By contrast, the MSD assay uses an antibody which, through rigorous epitope mapping, appears to detect only the full intact sclerostin molecule [14]. Thus, the Biomedica assay may overestimate true circulating sclerostin levels, which may at least in part explain why we found that sclerostin levels were, on average, 5- to 7-fold higher across groups of women when measured with the Biomedica assay as compared to the MSD assay. Importantly, these findings are consistent with a previous report [50]. Further, in contrast to the Biomedica assay results, we found no significant effect of aging on circulating sclerostin levels according to the MSD assay, which essentially mirrored the lack of changes in human bone *SOST* mRNA levels in the young versus old women (by both QPCR and RNAseq). In addition, according to both sclerostin assays, we found that E therapy in postmenopausal women reduced circulating sclerostin levels, which is not only consistent with previous studies [7,51], but also with the E-induced decrease in human bone *SOST* mRNA levels (by both QPCR and RNAseq). Collectively, these findings suggest that changes in serum sclerostin levels with aging and in response to E therapy, at least according to the MSD assay, reflect changes that occur in bone at the tissue level.

In addition to changes with aging, another goal of the present study was to utilize RNAseq analysis of human bone samples to interrogate potential gene targets that may be altered *in vivo* in response to E therapy. This analysis yielded a total of 21 genes (3 down- and 18 up-regulated, respectively) that were altered in E-treated women versus non-treated old women of a similar age. Interestingly, the expression *C19orf80* (coding for Betatrophin), which has roles in β-cell proliferation [52] and autophagy [53], decreased in response to E therapy. Further, it is noteworthy that the single most up-regulated gene (by 3.9-fold) in bone with E therapy, the orphan receptor *LGR5*, has been implicated in Wnt-β-catenin signaling [54], while the second most up-regulated gene (by 3.7-fold), *PPARGC1A*, is involved in mitochondrial biogenesis [55], antioxidant defense [56], and also integrates the mammalian clock with energy metabolism [57]. Thus, these E-regulated genes have critical functions in other non-skeletal tissues and disease processes, although elucidating their role(s) in the pathogenesis of bone fragility will require further work.

Our RNAseq analysis of human bone samples also revealed that *INHBB* expression decreased with aging but was restored to young adult levels in response to E therapy. It should be noted that the *INHBB*-encoded protein product can either homodimerize to form the activin B protein complex or heterodimerize with the *INHBA*-encoded protein product to form the activin AB protein complex [58]. While activins are known to be present at high levels in bone and likely play a major role in the regulation of bone metabolism [59], existing data are conflicting in that the effects of activins on bone formation and resorption are different among species and *in vitro/in vivo* models [60]. These findings may be explained by differences in cellular context, whereby the net effect of altered expression of mRNAs that encode for activins in bone, and its change in response to E, depends on the relative expression of activin pathway signaling components (e.g., receptors, follistatin antagonist, intracellular SMADs). Nevertheless, targeted inhibition of activin signaling *in vivo* using soluble activin type II receptors has been shown to enhance bone formation and inhibit bone resorption in both animals [61,62,63] and humans [64]. Thus, the potential biological significance of the decrease in *INHBB* mRNA levels with age and the restoration of these levels with E therapy remain to be more clearly defined.

One potential concern regarding RNAseq is validity of the technique. Beyond the fact that a number of the genes in bone that were significantly altered by RNAseq with aging or in response to E therapy were are also altered when assessed by QPCR [6,7], our direct comparison between RNAseq and QCPR showed a very strong correlation between the techniques, which provides additional ort for the high accuracy and reliability of RNAseq. Hence, we believe that RNAseq is a valid approach for transcriptional profiling. Given the advantages of RNAseq in that it provides an unbiased approach to profiling the entire transcriptome, application of this technique enabled us to identify several sets of genes and pathways altered in human bone biopsies with aging and in response to E therapy that were previously unrecognized. We do, however, acknowledge a limitation of our approach in that the changes we observed may not represent causality. Thus, some of the altered signaling pathways in bone with aging may not be the cause of bone loss, or alternatively could reflect compensatory changes that occur with aging. Clearly, further studies are needed to define the potential causal roles of the observed altered pathways in mediating bone loss.

A further limitation of our study is that the bone biopsies contain heterogeneous populations of cells, including bone marrow elements. As such, we do not know in which precise population(s) the observed changes in gene expression patterns are occurring, although we are in the process of untangling this complexity by using highly purified populations of osteoblasts and osteocytes from human bone biopsy samples [8]. This may also explain why we observed relatively few changes in the biopsies following E therapy, despite the major effects of E on bone metabolism. It is possible that changes occurring in bone cells (osteoblasts, osteocytes, or osteoclasts) following E therapy may have been dampened due to the heterogeneous nature of the biopsy samples, and future studies using more enriched cell populations may show a larger number of regulated genes in response to E. Alternatively, in future studies, RNAseq could be performed on marrow alone followed by subtracting from the bone plus marrow results, as previously described [65]. We should note, however, that the dramatic effects of E on bone (increase in PINP and decrease in CTX) may not necessarily require changes in a large number of genes. For example, we found that E reduced both bone sclerostin mRNA levels (by RNAseq and QPCR) and serum sclerostin levels (using 2 different commercial assays). This change in sclerostin production could well account for most of the effects of E on bone, given the marked effects of the sclerostin antibody in bone turnover and bone mass in humans [66], including very recent evidence that, in addition to inhibiting bone formation, sclerostin may also lead to an increase in bone resorption due to stimulation of RANKL production by osteocytes [67]. However, the identification of the further downstream gene expression changes following sclerostin inhibition by E will likely require more refined techniques, as noted above.

In conclusion, our findings demonstrate that aging alters a subset of the skeletal transcriptome, whereas E therapy appears to have significant, albeit less wide-ranging effects. To date, our results provide the most comprehensive assessment of the effects of aging and E therapy on the human skeleton’s gene expression profile, and serve as a valuable resource for the identification of novel biomarkers associated with age-related bone loss to better target therapies to specific patients. In addition, our study validates, in humans, several pathways associated with age-related bone loss in various animal models. Thus, the coordinated alteration of these pathways with aging emphasizes their potential importance in the pathogenesis of osteoporosis in humans. However, these signaling pathways are integrated with other networks and this overlap leads to enormous complexity which remains to be understood. Notwithstanding, interventions that modulate these pathways may lead to increased bone formation in humans and improved skeletal health.

## Supporting Information

S1 Fig QPCR confirms RNAseq data in human bone biopsies. Spearman’s correlation (*r* = 0.95) showing strong association of gene expression levels between RNAseq and QPCR in a subset of genes significantly (*p* < 0.05, *q* < 0.10) altered with aging (by RNAseq, n = 46).
(TIF)

S1 Protocol Mayo Clinic Institutional Review Board approved protocol for young versus old study. (PDF)

S2 Protocol Mayo Clinic Institutional Review Board approved protocol for estrogen study. (PDF)

S1 Table Genes regulated by aging in human bone. Complete gene list of the genes altered based on RNAseq analysis (median count ≥ 10, *p* < 0.05, false discovery rate [*q*] < 0.10) in human bone samples of young women relative to old women.
(XLSX)

S2 Table Genes regulated by short-term estrogen (STE) therapy in human bone. Complete gene list of the genes altered based on RNAseq analysis (median count ≥ 10, *p* < 0.05, false discovery rate [*q*] < 0.10) in bone samples of old women treated with STE therapy relative to old non-treated women.
(XLSX)

S3 Table Canonical pathways regulated by aging in human bone. Complete list of 73 significant (*p* < 0.05) pathways altered in human bone samples with aging by Ingenuity Pathway Analysis. Ratio refers to the ratio of genes altered in bone with aging divided by the total number of genes in that pathway.
(XLSX)

S4 Table Networks regulated by aging in human bone. Complete list of 25 networks altered in human bone samples with aging by Ingenuity Pathway Analysis, as well as corresponding diseases/functions, scores, and molecules.
(XLSX)

S5 Table Transcription factor binding sites regulated by aging in human bone. List of top 100 transcription factor binding sites of genes differentially expressed in the young versus. old dataset.
(XLSX)

S6 Table Genes associated with *LEF1* transcription factor binding sites. Lists of genes associated with two separate *LEF1* transcription factor binding sites: 1) TTGTTT (n = 93); and 2) CTTTGA (n = 60), as well as the 20 genes common to both lists.
(XLSX)

S7 Table Complete list of upstream regulators. List of all upstream regulators of genes altered with aging (median count ≥10, *q* < 0.10) in the young versus old dataset.
(XLSX)

S1 TREND Checklist TREND Statement Checklist. (PDF)
