# Supplementary figures and images for: Effects of Age and Estrogen on Skeletal Gene Expression in Humans as Assessed by RNA Sequencing
Source: PLoS One. 2015 Sep 24;10(9):e0138347. doi: 10.1371/journal.pone.0138347 (PMC4581624; doi:10.1371/journal.pone.0138347)

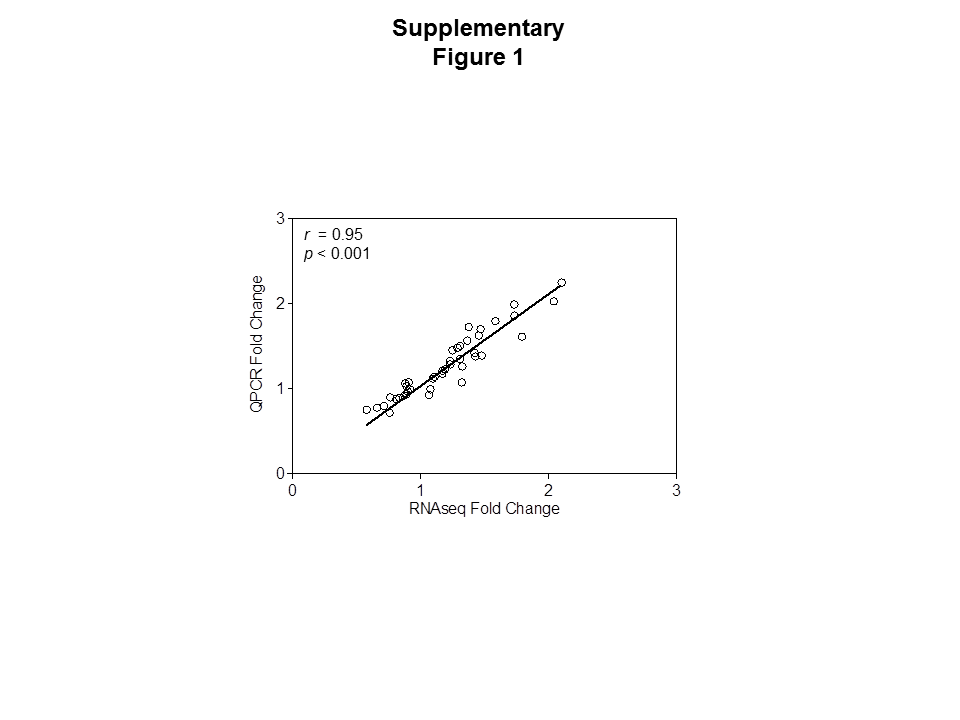

Supplement: S1 Fig — Spearman’s correlation (r = 0.95) showing strong association of gene expression levels between RNAseq and QPCR in a subset of genes significantly (p < 0.05, q < 0.10) altered with aging (by RNAseq, n = 46). (TIF) [file pone.0138347.s001.TIF]
