# Supplementary material for: Effects of Age and Estrogen on Skeletal Gene Expression in Humans as Assessed by RNA Sequencing
Source: PLoS One. 2015 Sep 24;10(9):e0138347. doi: 10.1371/journal.pone.0138347 (PMC4581624; doi:10.1371/journal.pone.0138347)
Supplement: S1 Protocol — (PDF) [file pone.0138347.s002.pdf]

MAYO CLINIC

IRB Protocol Template

Instructions for use of the protocol template:
1. Use the protocol template for original or working protocols.
2. Complete the form by tabbing through the fields or by clicking in each desired field.
3. When finished, save to your files.
4. For your IRBe application, open the application and upload the protocol template into the protocol field.

Instructions for revisions to the IRB-approved protocol in IRBe:
1. Open the protocol in IRBe, go to the Tools menu and select 'Track changes' from the Tools menu.
2. Make desired revisions (changes will be tracked).
3. When revisions are completed, save to your files (folder on your computer).
4. Upload the revised protocol (with tracked changes) into the protocol field of the IRBe modification form

Title: **Effects of Aging on Osteoprogenitor and Osteoblastic Cells**

Protocol Version/Date: 9/21/12 mod 9
IRB#: 10-007658
PI: Sundeep Khosla, M.D.

Abstract: Age-related bone loss and osteoporosis is an increasing public health problem. Understanding the mechanisms by which aging alters bone metabolism is critical for developing novel approaches to prevent and treat age-related osteoporosis. This is a working protocol for part of Aim 3 of our AG004875 PPG grant which was just renewed. We will focus on defining mechanisms for the age-related decrease in bone formation. We will use novel methods we have developed to examine gene expression in highly enriched bone marrow and bone osteoblastic cells. We will test whether the decrease in bone formation with aging is associated with with a decrease in markers of Wnt/BMP signaling and/or production and in othe genes related to bone formation by osteoblastic cells.

Schematic Design of the Study: We will study 2 groups of women (n = up to 30 per group), 30 of whom will be postmenopausal (Post) and age 65 years or older and 30 premenopausal (Pre) and meet the other inclusion/exclusion criteria described below. At baseline, both groups will have fasting, 8 am bloodwork drawn for bone formation (OCN and PINP) and resorption (CTx and TRAP 5b) markers. Three weeks later, both groups will have the fasting blood samples redrawn to assess stability in bone turnover markers and allow us to average the two time points in our analyses, additional peripheral blood will be drawn for the cell analyses, and bilateral bone marrow aspirates and biopsies will be performed for the analyses described below.
After completing some of the sample processing, we have found that we do not have enough RNA from some of the subjects to complete the aim. We would like to have these subjects repeat the study, if they are willing. We would re-consent them and repeat the screen labs as well as the remainder of the study visits. We would not use the data from their first visits, only the new data obtained from the second study visits.

Aims:
To test the hypothesis that aging is associated with a decrease in markers of Wnt/BMP signaling and/or production and in other genes related to bone formation in osteoblastic cells.

Methods

Description of Recruitment Methods:

How will subjects be identified?
Previous participants, volunteers from the community
How will subjects be contacted?
Mail, telephone, flyers, ad
Recruitment Materials (if applicable):
Letter, ads, flyers

Subject Population:

Number (total, each subgroup) up to 60, 30 per group
Gender:
Male 0

Female 40

Ages: Pre: 20-40 years; Post, 65 and older.

Inclusion Criteria:

Premenopausal subjects between 20 and 40 years of age, inclusive.

Post-menopausal subjects: At least 10 years postmenopausal; Menopause is defined as no menses for at least 1 year (or documented ovariectomy) and a serum FSH above 30 IU/L.

Exclusion Criteria:

1) Clinically significant abnormality in any of the following screening laboratory studies (to be reviewed and determined by PI or CI) : serum 25-hydroxyvitamin D (see below); phosphorus (minor change outside of normal guidelines is acceptable and does not impact the study); alkaline phosphatase and aspartate transaminase (AST) (minor change outside of normal guidelines is acceptable but not to exceed 50% above normal or ineligible); Creatinine (Cr) (minor change outside of normal guidelines is acceptable but not to exceed a value of 1.2 or ineligible); serum calcium must not exceed upper limits of normal guidelines or subject ineligible; For the Post group, FSH needs to be ≥30;TSH needs to be above 0.3 and not > 10; 2) Presence of significant liver disease, renal disease, malignancy (including breast cancer and myeloma), malabsorption syndrome, hypoparathyroidism, hyperparathyroidism, acromegaly, Cushing's syndrome, hypopituitarism, severe chronic obstructive pulmonary disease, untreated gallbladder disease, history of MI or stroke, or history of thrombophlebitis or deep venous thrombosis; 3) Undergoing treatment with any of the following drugs: adrenocorticoid steroids (3 months or longer at anytime or > 10 days of treatment within the previous 12 months), anticonvulsant therapy (within the previous year), sodium fluoride (any history of treatment with fluoride), pharmacological doses of thyroid hormone (causing decline of thyroid stimulating hormone [TSH] below normal), calcium supplementation of more than 1200 mg/d (within the preceding 3 months), bisphosphonates in the past 3 years, calcitonin (within the past six months), E therapy or treatment with a selective estrogen receptor modulator (within the past 6 months), PTH use in the past. Subjects with a clinical history of any fractures in the previous three years will also be excluded. Pre-menopausal women who are on oral contraceptives will be acceptable for study, except for women on progesterone only contraception (since estrogen levels are reduced in this group).

Step-by-Step Schedule (include all procedures, therapies – attach a table or flow chart if there are multiple procedures or visits and indicate the window of number of days in which the participant may return for follow-up visits):

Screening laboratory studies will be performed as outpatients in the CRU at either Charlton 7 or Domitilla 5, Saint Marys Hospital. If a subject is found to have low body stores of Vitamin D as assessed by the serum 25-hydroxyvitamin D of <20 ng/ml, they will be treated with 1000 units/day of Vitamin D for 8 weeks and then have their level rechecked. If the level is still < 20 ng/ml, they will receive a second course of treatment; if the level is still < 20 ng/ml after the second course, they will not continue in the study and will be referred to their primary physician for further evaluation. If it is 20 ng/ml or greater they will then continue with the study.

We will study 2 groups of women (n = up to 30 per group), 30 of whom will be postmenopausal (Post) and age 65 years or older and 30 premenopausal (Pre) and meet the other inclusion/exclusion criteria described below. At baseline, both groups will have fasting, 8 am bloodwork drawn for bone formation (OCN and PINP) and resorption (CTx and TRAP 5b) markers. Three weeks later, both groups will have the fasting blood samples redrawn to assess stability in bone turnover markers and allow us to average the two time points in our analyses, additional peripheral blood will be drawn for the cell analyses, and bone marrow aspirates and biopsies will be performed for the analyses described below.

Biospecimen (types, number, volume, processing, storage):

Screening visit: blood sample for the following-CBC, serum calcium, phosphorus, alkaline phosphatase, creatinine, AST, FSH, 25(OH)D, and TSH.

Baseline: blood draw of 50 ml for bone formation markers (OCN and PINP) and bone resorption markers (CTx and TRAP 5b)

3 weeks after baseline draw: urine pregnancy test (Women who are status post hysterectomy, tubal ligation or post menopausal do not need pg test); blood draw of up to 250 ml for bone formation markers (OCN and PINP) and bone resorption markers (CTx and TRAP 5b) and cells for RNA

bone marrow aspirate and biopsy: These procedures are as routinely performed clinically in Hematology. These will be performed by the Transfusion Therapy Center (TTC) nurses or our study coordinator, Louise McCready, RN, who has been trained by the TTC nurses, under the supervision of Dr. Khosla. This will provide up to 80 ml of marrow. 2 ml of fresh, unprocessed bone marrow will be used for analysis of apoptosis. The remaining bone marrow aspirate and all of the peripheral blood will be depleted of the red blood cells and most of the platelets and granulocytes by density-gradient centrifugation over Ficoll-Paque. The mononuclear cells (MNCs) will then be harvested from the interface, washed, and cell number and cell viability with trypan blue will be measured. Following Ficoll extraction, an aliquot of 4 million cells will be removed from the bone marrow sample for flow cytometry for lin-/AP+ cells co-stained with phospho-GSK-3β and phospho-Smad1/5 antibodies. An additional aliquot of 10 million cells will be removed and CD14+ cells isolated by MACS. The remaining MNCs obtained from bone marrow and peripheral blood will be magnetically labeled with the human lineage cell depletion kit (Miltenyi Biotec GmbH) and the cell suspension will be loaded onto an autoMACS cell sorter (Miltenyi Biotec GmbH, Germany). Following hematopoietic lineage (lin) depletion, immunofluorescent staining of lin- bone marrow and peripheral blood cells will be performed using a biotinylated anti-human AP monoclonal antibody and an isotype matched control, both from R & D Systems. The lin-/AP+ cell population from bone marrow and peripheral blood MNCs will be sorted using FACS sorting (BD FACSAria Cell-Sorting System). FACS sorted lin-/AP+ cells from bone marrow and peripheral blood will be stored in RLT buffer at -80º C for later extraction of RNA.

The biopsies, which contain cortical and trabecular bone, will be processed in our laboratory for analysis of gene expression as for the bone marrow cells.

Serum OCN will be measured by the ELSA-Osteo two-site IRMA (Cisbio-US, interassay CV < 8%); serum PINP will be measured by RIA (Orion Diagnostica, interassay CV < 9%); serum CTx will be measured by a one-step ELISA kit (Nordic Bioscience Diagnostics, inter-assay CV < 8%); and TRAP 5b will be measured by ELISA (Immunodiagnostic Systems Ltd., inter-assay CV <14%).

Plan for Dose Modification if Toxicity occurs (if applicable):

Statistical Considerations

Endpoints

Primary: Primary: Our primary endpoints will be differences between Pre and Post women in (1) Wnt and BMP target genes; (2) osteoblast vs. adipocyte commitment genes and the genes related to osteoblast differentiation; (3) apoptosis genes; and (4) the percentage of apoptotic lin-/AP+ cells as quantified by flow cytometry.

Secondary:

Power Statement:

Based on our analysis of 6 postmenopausal women in whom we analyzed 128 genes simultaneously using our in-house QPCR arrays, we estimate that with data from 20 subjects per group, we would have 80-90% power to detect differences of 2-fold or less for 61% of the genes analyzed, 2-3 fold differences for 30% of the genes, and > 3-fold differences for 9% of the genes using a two-sample t-test with a significance level of 0.05. Thus, we should (on average) have excellent power to detect under 2-fold changes in gene expression for approximately 2/3 of the genes analyzed and moderate power for approximately 1/3 of the genes. We should note that in our Preliminary Study in men, we found greater than 10-fold higher Lef1 mRNA levels in the osteoblastic cells from sex steroid replete as compared to sex steroid deficient men, so we expect fairly large changes in some of the pathways being examined. Our primary statistical approach will be the two-sample t-test, using non-parametric approaches if necessary. We will also explore a modification of Gene Set Enrichment Analysis **(GSEA)** to assess the significance of pre-defined gene-sets, rather than individual genes. In other settings, this approach has increased statistical power by borrowing strength from across the gene-set.

Based on similar power calculations for the Annexin V assay (see Preliminary Studies), we estimate that with data from 20 subjects per group, we should have 90% power to detect a 1.8-fold decrease in the percentage of lin-/AP+ cells undergoing apoptosis using a two-sample t-test with a significance level of 0.05. Studies in mice have demonstrated that E deficiency is associated with a doubling in the percentage of

osteoblasts undergoing apoptosis, so we should have excellent power to see even smaller increases in humans.

Data Analysis:
See above

Protocol form, page 2

Human Safety Aspects

Risks Venipuncture: The risks of venipuncture for blood drawing include pain, bleeding, bruising, infection and inflammation at the site. The total amount of blood withdrawn will not exceed 550 ml over an eight week period. Hemoglobin measurements at the screen visit must be greater than 11.5 g in females in order to participate. All subjects will refrain from giving blood or being on other research studies for eight weeks prior to the study, during the study, and for 8 weeks after completion of the study.

Intravenous access: The risks of intravenous access are as above for venipuncture. There is also the potential risk for blood borne infection through the catheter site.

Bone marrow aspirates and biopsies: These are routine procedures associated with minimal or no complications. Possible side effects include pain, bleeding, bruising and infection at the site where the bone marrow is removed. Pain is minimized by the use of local anesthesia, and moderate sedation is offered to the subjects. Bleeding at the site may occur at the time of procedure; to minimize this, pressure is applied to the area until bleeding stops. This can be a painful procedure, and the discomfort may last for several days.

Medications used:

Vitamin D has little risk. Side effects that may occur are abdominal cramping, headache weight gain, nausea, vomiting and constipation.

Moderation sedation: Possible complications include drowsiness, a fall in blood pressure, or a slowing of the breathing rate. Minor complications may also include fainting, nausea, or vomiting. A urine pregnancy test will also be done prior to start of procedure.

Individual Subject Stopping Rules (if applicable):

DSMB (if applicable):

Members:

Charter:

Stopping Rules for Efficacy and Safety (if applicable):

Questionnaires that ask about Depression (if applicable)

Included in study? Yes ☐ No X☐

If “Yes”, state the plan of management for subjects with possible depression:
