# Supplementary material for: Effects of Age and Estrogen on Skeletal Gene Expression in Humans as Assessed by RNA Sequencing
Source: PLoS One. 2015 Sep 24;10(9):e0138347. doi: 10.1371/journal.pone.0138347 (PMC4581624; doi:10.1371/journal.pone.0138347)
Supplement: S2 Protocol — (PDF) [file pone.0138347.s003.pdf]

MAYO CLINIC

IRB Protocol Template

Instructions for use of the protocol template:
1. Use the protocol template for original or working protocols.
2. Complete the form by tabbing through the fields or by clicking in each desired field.
3. When finished, save to your files.
4. For your IRBe application, open the application and upload the protocol template into the protocol field.

Instructions for revisions to the IRB-approved protocol in IRBe:
1. Open the protocol in IRBe, go to the Tools menu and select 'unprotect' from the list.
2. Select 'Track changes' from the Tools menu. The protocol is now ready to make changes.
3. When revisions are completed, save to your files.
4. Upload the revised protocol into the protocol field of the IRBe application.

Title: Effects of Acute Estrogen Therapy on Bone Formation
Protocol Version/Date: #3 **Oct/2011**

IRB#: **09-001935**

PI: Sundeep Khosla, MD

Abstract:
Estrogen (E) deficiency is the major cause of postmenopausal osteoporosis. Understanding the mechanisms by which E regulates bone metabolism is critical for developing novel approaches to prevent and treat this disorder. This is a working protocol for Aim 2 of our AG004875 PPG grant which was just renewed. We will focus on defining mechanisms for the age-related decrease in bone formation and the role of E deficiency in mediating this decrease. We will use novel methods we have developed to examine gene expression in highly purified bone marrow osteoblastic cells. We will test whether the increase in bone formation previously observed following acute E treatment in women is associated with an increase in markers of Wnt/BMP signaling and/or production and in othe genes related to bone formation by osteoblastic cells.

Schematic Design of the Study: **We will study 20 post-menopausal women, all of whom will be at least 10 years postmenopausal (in order to avoid confounding effects of early postmenopausal changes in bone turnover) and meet the other inclusion/exclusion criteria described below. Subjects will be treated with estrogen for 3 weeks. If the subjects have not had a negative mammogram in the past year, we will obtain a digital screening mammogram and document a negative mammogram prior to entering them into the study. The women will be treated with a transdermal E2 patch (0.1 mg/d) for 3 weeks. At baseline, they will have fasting, 8 am bloodwork drawn for bone formation (OCN and PINP) and resorption (CTx and TRAP 5b) markers. Three weeks later, the will have the fasting blood samples redrawn to assess changes in bone turnover following E treatment, additional peripheral blood will be drawn for the cell analyses, and a bone marrow aspirate and biopsy performed for the analyses described below.**

Aims: In an experimental paradigm of acute (3 weeks) E treatment, test the hypothesis that during the acute uncoupling phase, when bone formation is increased, there is an increase in markers of Wnt/BMP signaling and/or production and in other genes related to bone formation in osteoblastic cells.

Methods

Description of Recruitment Methods:

How will patients be identified?
previous participants, volunteers from community
How will patients be contacted?
mail, telephone, flyers, ad

Recruitment Materials (if applicable):
letter, ads, flyers

Patient Population:

Number (total, each subgroup) 20

Gender:

Male 0

Female 20

Ages**:** 65 and older

Inclusion Criteria:

At least 10 years postmenopausal; Menopause is defined as no menses for at least 1 year (or documented ovariectomy) and a serum FSH above 30 IU/L.

Exclusion Criteria:

1) Clinically significant abnormality in any of the following screening laboratory studies (to be reviewed and determined by PI or CI) : serum 25-hydroxyvitamin D (see below); phosphorus (minor change outside of normal guidelines is acceptable and does not impact the study); alkaline phosphatase and aspartate transaminase (AST) (minor change outside of normal guidelines is acceptable but not to exceed 50% above normal or ineligible); Creatinine (Cr) (minor change outside of normal guidelines is acceptable but not to exceed a value of 1.2 or ineligible); serum calcium must not exceed upper limits of normal guidelines or subject ineligible; FSH needs to be ≥30;TSH needs to be above 0.3 and not > 10; 2) Presence of significant liver disease, renal disease, malignancy (including breast cancer and myeloma), malabsorption syndrome, hypoparathyroidism, hyperparathyroidism, acromegaly, Cushing's syndrome, hypopituitarism, severe chronic obstructive pulmonary disease, untreated gallbladder disease, history of MI or stroke, or history of thrombophlebitis or deep venous thrombosis; 3) Undergoing treatment with any of the following drugs: adrenocorticoid steroids (3 months or longer at anytime or > 10 days of treatment within the previous 12 months), anticonvulsant therapy (within the previous year), sodium fluoride (any history of treatment with fluoride), pharmacological doses of thyroid hormone (causing decline of thyroid stimulating hormone [TSH] below normal), calcium supplementation of more than 1200 mg/d (within the preceding 3 months), bisphosphonates in the past, calcitonin (within the past six months), E therapy or treatment with a selective estrogen receptor modulator (within the past 6 months), PTH use in the past. Subjects with a clinical history of an osteoporotic fracture (vertebral, hip, or distal forearm) within the previous 3 years will also be excluded.

Step-by-Step Schedule

Screening laboratory studies will be performed as outpatients in the CRU at either Charlton 7 or Domitilla 5, Saint Marys Hospital. If a subject is found to have low body stores of Vitamin D as assessed by the serum 25-hydroxyvitamin D of <20 ng/ml, they will be treated with 1000 units/day of Vitamin D for 8 weeks and then have their level rechecked. If the level is still < 20 ng/ml, they will receive a second course of treatment; if the level is still < 20 ng/ml after the second course, they will not continue in the study and will be referred to their primary physician for further evaluation. If it is 20 ng/ml or greater they will then continue with the study.

If they have not had a negative mammogram in the past year, they will undergo a screening mammogram. A negative mammogram is one that is read as such by the radiologist, although minor abnormalities such as fibrocystic disease, benign calcifications, etc which are judged by the radiologist as benign changes will not preclude the subjects from participating.

We will study 20 post-menopausal women, all of whom will be at least 10 years postmenopausal (in order to avoid confounding effects of early postmenopausal changes in bone turnover) and meet the other inclusion/exclusion criteria described below. If the subjects have not had a negative mammogram in the past year, we will obtain a digital screening mammogram and document a negative mammogram prior to entering them into the study. The participants will be treated with a transdermal E2 patch (0.1 mg/d) for 3 weeks. At baseline, they will have fasting, 8 am bloodwork drawn for bone formation (OCN and PINP) and resorption (CTx and TRAP 5b) markers. Three weeks

later, they will have the fasting blood samples redrawn to assess changes in bone turnover following E treatment, additional peripheral blood will be drawn for the cell analyses, and bilateral bone marrow aspirate and biopsy performed for the analyses described below.

Biospecimen (types, number, volume, processing, storage):

Screening visit: blood sample for the following-CBC, serum calcium, phosphorus, alkaline phosphatase, creatinine, AST, FSH, 25(OH)D, and TSH.

Baseline: blood draw of 50 ml for bone formation markers (OCN and PINP) and bone resorption markers (CTx and TRAP 5b)

3 weeks after baseline draw: blood draw of 50 ml for bone formation markers (OCN and PINP) and bone resorption markers (CTx and TRAP 5b) and cells for RNA

Bilateral bone marrow aspirate and biopsy: These procedures are as routinely performed clinically in Hematology. These will be performed by the Transfusion Therapy Center (TTC) nurses or our study coordinator, Louise McCready, RN, who has been trained by the TTC nurses, under the supervision of Dr. Khosla. This will provide up to 80 ml of marrow. 2 ml of fresh, unprocessed bone marrow will be used for analysis of apoptosis. The remaining bone marrow aspirate and all of the peripheral blood will be depleted of the red blood cells and most of the platelets and granulocytes by density-gradient centrifugation over Ficoll-Paque. The mononuclear cells (MNCs) will then be harvested from the interface, washed, and cell number and cell viability with trypan blue will be measured. Following Ficoll extraction, an aliquot of 4 million cells will be removed from the bone marrow sample for flow cytometry for lin-/AP+ cells co-stained with phospho-GSK-3β and phospho-Smad1/5 antibodies. An additional aliquot of 10 million cells will be removed and CD14+ cells isolated by MACS. The remaining MNCs obtained from bone marrow and peripheral blood will be magnetically labeled with the human lineage cell depletion kit (Miltenyi Biotec GmbH) and the cell suspension will be loaded onto an autoMACS cell sorter (Miltenyi Biotec GmbH, Germany). Following hematopoietic lineage (lin) depletion, immunofluorescent staining of lin- bone marrow and peripheral blood cells will be performed using a biotinylated anti-human AP monoclonal antibody and an isotype matched control, both from R & D Systems. The lin-/AP+ cell population from bone marrow and peripheral blood MNCs will be sorted using FACS sorting (BD FACSAria Cell-Sorting System). FACS sorted lin-/AP+ cells from bone marrow and peripheral blood will be stored in RLT buffer at -80º C for later extraction of RNA.

The biopsies, which contain cortical and trabecular bone, will be processed in our laboratory for analysis of gene expression as for the bone marrow cells.

Serum OCN will be measured by the ELSA-Osteo two-site IRMA (Cisbio-US, interassay CV < 8%); serum PINP will be measured by RIA (Orion Diagnostica, interassay CV < 9%); serum CTx will be measured by a one-step ELISA kit (Nordic Bioscience Diagnostics, inter-assay CV < 8%); and TRAP 5b will be measured by ELISA (Immunodiagnostic Systems Ltd., inter-assay CV <14%).

Plan for Dose Modification if Toxicity occurs (if applicable):

Statistical Considerations

Endpoints

Primary: Our primary endpoints will be differences between the E+ women in this protocol compared to E- post-menopausal women who participated in IRB 10-007658 in (1) Wnt and BMP target genes; (2) osteoblast vs. adipocyte commitment genes and the genes related to osteoblast differentiation; (3) apoptosis genes; and (4) the percentage of apoptotic lin-/AP+ cells as quantified by flow cytometry.

Secondary:

Power Statement:

Based on our analysis of 6 postmenopausal women in whom we analyzed 128 genes simultaneously using our in-house QPCR arrays, we estimate that with 20 subjects in this study and the data from the 20 subjects

in IRB 10-007658, we would have 80-90% power to detect differences of 2-fold or less for 61% of the genes analyzed, 2-3 fold differences for 30% of the genes, and > 3-fold differences for 9% of the genes using a two-sample t-test with a significance level of 0.05. Thus, we should (on average) have excellent power to detect under 2-fold changes in gene expression for approximately 2/3 of the genes analyzed and moderate power for approximately 1/3 of the genes. We should note that in our Preliminary Study in men, we found greater than 10-fold higher Lef1 mRNA levels in the osteoblastic cells from sex steroid replete as compared to sex steroid deficient men, so we expect fairly large changes in some of the pathways being examined. Our primary statistical approach will be the two-sample t-test, using non-parametric approaches if necessary. We will also explore a modification of Gene Set Enrichment Analysis **(GSEA)** to assess the significance of pre-defined gene-sets, rather than individual genes. In other settings, this approach has increased statistical power by borrowing strength from across the gene-set. Details regarding this analysis are included in Core A.

Based on similar power calculations for the Annexin V assay (see Preliminary Studies), we estimate that with 20 subjects per group, we should have 90% power to detect a 1.8-fold decrease in the percentage of lin-/AP+ cells undergoing apoptosis using a two-sample t-test with a significance level of 0.05. Studies in mice have demonstrated that E deficiency is associated with a doubling in the percentage of osteoblasts undergoing apoptosis, so we should have excellent power to see even smaller increases in humans.

Data Analysis:
See above

Human Safety Aspects

Risks: Venipuncture: The risks of venipuncture for blood drawing include pain, bleeding, bruising, infection and inflammation at the site. The total amount of blood withdrawn will not exceed 550 ml over an eight week period. Hemoglobin measurements at the screen visit must be greater than 11.5 g in females in order to participate. All subjects will refrain from giving blood or being on other research studies for eight weeks prior to the study, during the study, and for 8 weeks after completion of the study.
Intravenous access: The risks of intravenous access are as above for venipuncture. There is also the potential risk for blood borne infection through the catheter site.

Bone marrow aspirates and biopsies: These are routine procedures associated with minimal or no complications. Possible side effects include pain, bleeding, bruising and infection at the site where the bone marrow is removed. Pain is minimized by the use of local anesthesia, and moderate sedation is offered to the subjects. Bleeding at the site may occur at the time of procedure; to minimize this, pressure is applied to the area until bleeding stops. This can be a painful procedure, and the discomfort may last for several days.

Medications used:

Estradiol dermal patch is used at a dose that approximates normal circulating estradiol levels in premenopausal women and will be administered for only 3 weeks. The patches may cause a topical allergic type reaction. This may cause vaginal bleeding in women with intact uteri. However, it has been our experience in a number of other studies that for postmenopausal women who have been amenorrheic for >1 yr that bleeding is uncommon when E administration is given for only 3 weeks. Other E related symptoms such as mastodynia, premenstrual syndrome symptoms, and pedal edema are very unlikely with such a short period of treatment. The Women's Health Initiative, a large prospective trial of an average of 5.2 yrs with Prempro reported the following adverse effects (difference from controls): heart attacks, 7 per yr per 10,000 patients studied; stroke, 8 per yr per 10,000 patients studied; venous thromboembolism, 8 per yr per 10,000 patients studied; breast cancer, 8 per yr per 10,000 patients studied. About 25% of American postmenopausal women are still taking E therapy chronically, and we do not believe that this short exposure will prove to produce problems. Nonetheless, the study nurse will remain in regular contact with the study subjects and any adverse effects will be investigated.

Vitamin D has little risk. Side effects that may occur are abdominal cramping, headache weight gain, nausea, vomiting, constipation.

Moderation sedation: Possible complications include drowsiness, a fall in blood pressure, or a slowing of the breathing rate. Minor complications may also include fainting, nausea, or vomiting.

Radiation: The amount of radiation they will receive has a low risk of harmful effects.

Individual Patient Stopping Rules (if applicable):
DSMB (if applicable): NA
Members:

Charter:

Stopping Rules for Efficacy and Safety (if applicable):

Questionnaires that ask about Depression (if applicable)
Included in study? Yes ☐ No ☒
If "Yes", state the plan of management for subjects with possible depression:

When submitting this form to IRBe, attach a copy of the budget and consent form. Thank you.
